# Absolute Sustainability Assessment of Flue Gas Valorization to Ammonia and Synthetic Natural Gas

**DOI:** 10.1021/acssuschemeng.3c05246

**Published:** 2023-12-08

**Authors:** Sebastiano Carlo D’Angelo, Julian Mache, Gonzalo Guillén-Gosálbez

**Affiliations:** Department of Chemistry and Applied Biosciences, Institute for Chemical and Bioengineering, ETH Zurich, Vladimir-Prelog-Weg 1, Zurich 8093, Switzerland

**Keywords:** postcombustion capture, carbon capture and utilization, LCA, planetary boundaries, renewables, techno-economic analysis

## Abstract

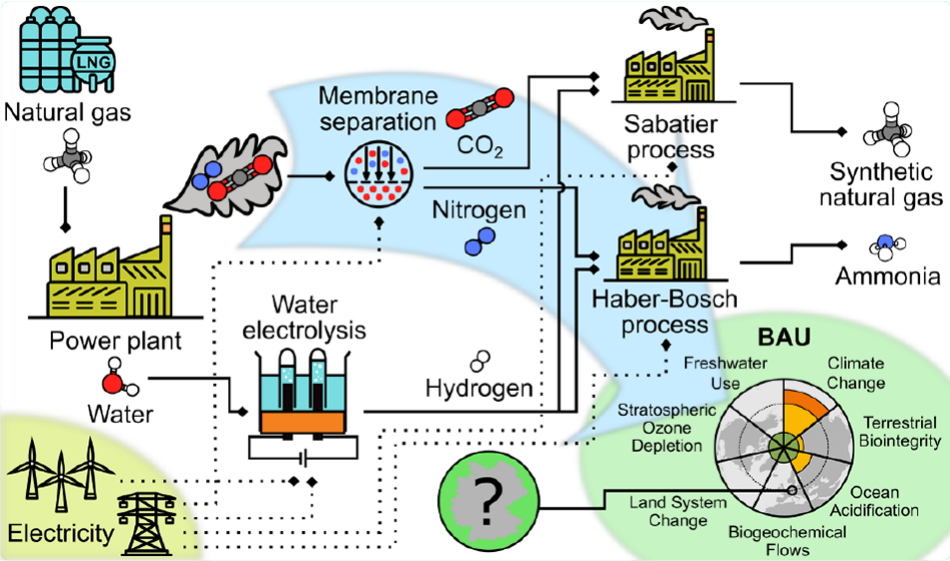

Carbon capture and utilization has gained attention to potentially curb CO_2_ emissions while generating valuable chemicals. These technologies will coexist with fossil analogs, creating synergies to leverage circular economy principles. In this context, flue gas valorization from power plants can assist in the transition. Here, we assessed the absolute sustainability of a simulated integrated facility producing ammonia and synthetic natural gas from flue gas from a combined-cycle natural gas power plant based in Germany, using hydrogen from three water electrolysis technologies (proton exchange membrane, alkaline, and solid oxide cells), nitrogen, and CO_2_. For the first time, we applied the planetary boundaries (PBs) framework to a circular integrated system, evaluating its performance relative to the safe operating space. The PB-LCA assessment showed that the alternative technologies could significantly reduce, among others, the impact on climate change and biosphere integrity when compared to their fossil counterparts, which could be deemed unsustainable in climate change. Nevertheless, these alternative technologies could also lead to burden shifting and are not yet economically viable. Overall, the investigated process could smoothen the transition toward low-carbon technologies, but its potential collateral damages should be carefully considered. Furthermore, the application of the PBs provides an appealing framework to quantify the absolute sustainability level of integrated circular systems.

## Abstract 2

An advanced environmental and economic assessment is applied to a process valorizing flue gas from natural gas power plants.

## Introduction

A crucial step in the sustainable transition of the energy and chemical industries is reaching the independence of power generation from fossil fuels. However, in this transition phase, greenhouse gas (GHG) emissions from power plants still under operation will have to be curbed. In addition to this, a circular economy mindset should be adopted to minimize wastes,^1^ thus maximizing the efficient use of natural resources and valorizing side outputs into valuable materials that can re-enter the economy. In this context, carbon capture and utilization (CCU) is gaining increasing interest in the scientific community. Specifically, carbon dioxide (CO_2_) from point sources or from direct air capture could be transformed into added value chemicals that could displace fossil-based production of the same products. Such production, if powered by environmentally friendly energy sources, could lead to carbon neutral or even carbon negative emissions for such chemicals on a cradle-to-gate basis.^2^

Considering the above, here we shall investigate flue gas valorization from power plants, which could be a viable technology to achieve significant GHG emission reductions while applying circular thinking. Among the main advantages, by revalorizing the captured CO_2_ back to methane, thanks to the use of processes such as the Sabatier reaction,^3,4^ electrochemical CO_2_ reduction,^5^ or biodigesters,^6^ it would be possible to take further advantage of the natural gas infrastructure already in place in most of the developed countries, without significant additional costs of distribution.^7^ In addition to this, the remaining components of the purified flue gas, consisting mainly of reasonably pure nitrogen (N_2_), could be also combined with renewably produced hydrogen (H_2_) and valorized to products such as ammonia (NH_3_), regarded as a potential alternative energy storage vector in a future decarbonized economy.^8^ Both these routes are regarded as expressions of the concept of power-to-X when, in particular, H_2_ used for the reactions is produced electrocatalytically from renewable power and could serve as an alternative avenue to store the excess electricity produced in peak times from intermittent sources.^9^

Recent work by Castellani et al.^10^ proposed an integrated process that separates flue gas from power plants into its main components: CO_2_ and N_2_. These streams are then upgraded into synthetic natural gas (SNG) and NH_3_ through the Sabatier and the Haber–Bosch (HB) process, respectively, using electrolytic H_2_. This concept shows several advantages. First, what was originally a waste stream could be upgraded to promising low-carbon energy vectors. Second, the produced SNG could reduce the consumption of natural gas power plants, in line with circular thinking. Third, by producing H_2_ from intermittent energy sources such as wind or solar power, part of the excess electricity during peak power generation times could be absorbed avoiding curtailment.^11^

From an economic perspective, similar power-to-X concepts, focusing solely on power-to-SNG^12−15^ and power-to-NH_3_^16,17^ separately, had highlighted how, while such approaches are not yet competitive with their fossil counterparts, they might soon close the gap, thanks to technical improvements in water electrolysis units and cheaper electricity costs.

The environmental impact of these emerging technologies has been estimated through life cycle assessment (LCA), evaluating their sustainability along the whole supply chain. Several works applied LCA on NH_3_ and SNG production through many alternative pathways to the business-as-usual (BAU).^10,18,19^ However, these studies often focus on carbon footprint solely while neglecting other impact categories.^18,19^ This is a major shortcoming, as the occurrence of burden shifting (one environmental category improves at the expense of exacerbating others) has been reported in several CCU routes.^20−23^

The main limitation of standard LCA metrics is that they are suitable for comparison purposes but provide little insight into whether a specific system is sustainable in absolute terms, i.e., relative to the ecological capacity of the entire Earth system. In the past years, absolute sustainability assessments emerged to overcome this limitation, which are based on the recently proposed planetary boundaries (PBs) concept^24^ that establishes a set of critical thresholds on key Earth-system processes, which should never be exceeded. Transgressing the PBs could shift the Planet’s current state, challenging the Earth’s resilience. These limits, taken as a whole, define a safe operating space (SOS) within which anthropogenic activities should lie. Studies incorporating the PBs in chemical and fuel assessments are scarce and started to emerge only recently.^25−27^

In this work, we apply a PB-LCA methodology to evaluate valorization pathways of flue gas from a natural gas power plant to produce NH_3_ and SNG using renewable H_2_. In particular, we quantify the transgression levels relative to the SOS, focusing on a valorization system located in Germany and comparing the results with the fossil-based analogs. In addition, impacts on human health are assessed to complement the picture provided by the PBs methodology. To the best of the authors’ knowledge, this is the first time that such a novel methodology for environmental assessments has been applied to the proposed process.

## Methods

### Process Description

**Figure 1:**
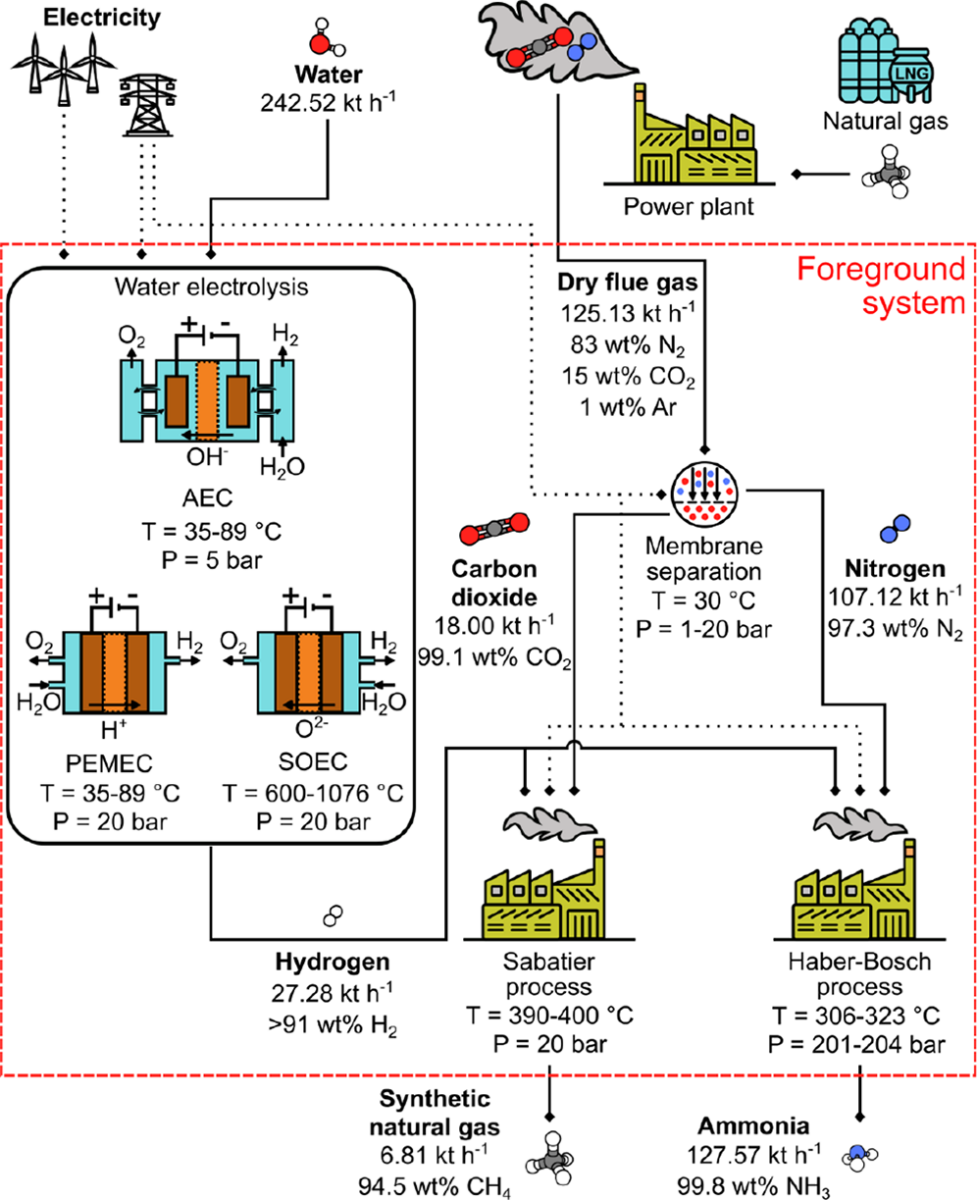
Conceptual schematic of the assessed process. The sections explicitly modeled in the analysis are enclosed by a red dashed rectangle. The acronyms for the water electrolysis units are as follows: AEC: alkaline electrolytic cell; PEMEC: proton exchange membrane electrolytic cell; and SOEC: solid oxide electrolytic cell. The main operating conditions and stream flows from the reference simulations used are reported. Additional information on the process steps can be found in Section 2 in the Supporting Information.

The block flow diagram of the process is presented in Figure 1. It encompasses four main stages: membrane separation, water electrolysis, the Sabatier process, and the HB process. Flue gas, fed as the input to the valorization system at 200 °C and 20 bar, was assumed to originate from a combined-cycle natural gas power plant with a flue gas recirculation factor of 0.42.^10,28^ The wet gas composition is summarized in Table S2 in the Supporting Information. The flue gas is stored before being fed to the process to allow continuous operation of the valorization plant even when the power output of the upstream system might be variable. Other types of fossil-based power plants, such as coal-based systems, show different compositions, usually with higher shares of CO_2_ content.^29^ However, with fine-tuning of the CO_2_ capture unit, it would be possible to adapt the analysis to other flue gas sources, as the impacts might vary almost directly in proportion to the main shares of the two main components to valorize, i.e., CO_2_ and N_2_.

H_2_ production is modeled in Aspen Custom Modeler (ACM) using three different scenario types associated with different technologies: proton exchange membrane (PEMEC),^30^ alkaline electrolytic cell (AEC),^31^ and solid oxide electrolytic cell (SOEC)^32^. Additional modeling details are included in Section 2.2 in the Supporting Information.

Before being fed into the membrane separation system, the oxygen (O_2_) impurities present in the flue gas are catalytically reduced to water using H_2_ in stoichiometric amounts in an isothermal equilibrium reactor at 350 °C. To avoid condensation in the membrane units, the flue gas is dried before entering the membrane assembly. First, the flue gas is cooled to 30 °C and flashed. Subsequently, triethylene glycol (TEG) dehydration was introduced under the same outlet conditions of the flash.

The flue gas is then fed to a four-stage membrane separation, whose membrane model was built in ACM based on previous work.^33^ At the permeate side, operating in the range 1–1.5 bar, a N_2_-enriched stream is obtained, while at the retentate side, working with a fixed pressure of 20 bar, a CO_2_-rich stream is separated. The process parameters were selected to obtain output streams with above 97% mass purity of N_2_ and CO_2_ at the permeate and retentate, respectively. Further details about the ordinary differential equation system solved in ACM, the full list of parameters used in the simulation, and additional insights on the membrane assembly configuration are included in Section 2.1 in the Supporting Information.

For the Sabatier reaction, kinetics were implemented according to the work by Rönsch et al.^34^ The CO_2_-rich stream is compressed to 20 bar and mixed with H_2_. After this step, the mixture is fed to a first isothermal reactor working at 390 °C. The reactor outlet is cooled to 30 °C, and water removal takes place in a flash before feeding the mixture to a second isothermal reactor working at 400 °C. The outlet from this unit again undergoes water removal in a fashion similar to the outlet of the first reactor, and finally, the last water impurities are removed through TEG dehydration. The produced output meets pipeline specifications in terms of impurities.^35,36^

The Sabatier process is tolerant to N_2_ impurities, since N_2_ behaves as an inert gas. In contrast, CO_2_ impurities should not be fed into the HB process. Consequently, as for the industrial standard, a purification step converting all the CO_2_ to methane in the N_2_-rich stream through methanation was included.^16^ Accordingly, H_2_ is fed in a stoichiometric ratio relative to CO_2_ to consume it entirely. Additional details about the kinetics of the methanation reactor can be found in Section 2.3 in the Supporting Information.

Finally, for NH_3_, a detailed model in Aspen HYSYS was developed based on a standard HB industrial process, based on D’Angelo et al.^16^ and considering an N_2_ conversion of 98.6%. Additional details can be found in Section 2.4 in the Supporting Information.

### Life Cycle Assessment and Planetary Boundaries Analysis

The LCA was performed following the ISO 14040 and 14044.^37,38^

In the first LCA phase, the goal and scope definition, the scenarios, and their associated assumptions were selected. Regarding the proposed alternative process, two different typologies of scenarios were considered, differing in their electricity source. All the scenarios assume that electricity powering the membrane separation, N_2_ purification, Sabatier process, and HB process must be nonintermittent to ensure a smooth operation of the processes, which are mainly relying on thermal inputs. Accordingly, these steps are powered by the German 2020 power grid mix.^39^ At the same time, one scenario type assumes that water electrolysis is powered by the same mix, while the second type uses wind energy for the water-splitting step. Moreover, three different types of water electrolysis units were assumed and compared: PEMEC, AEC, and SOEC. PEMEC was taken as the base case, since it is currently considered the most suitable for intermittent operation.^11^ Hence, six scenarios were assessed here for the proposed valorization process: one scenario using PEMEC H_2_ powered by grid electricity (PEMEC-Grid) and by wind energy (PEMEC-Wind), the same scenarios but using AEC (AEC-Grid and AEC-Wind), and a third pair of scenarios using SOEC (SOEC-Grid and SOEC-Wind). A cradle-to-gate study was adopted to quantify the absolute sustainability level of the proposed flue gas valorization routes and an equivalent functional unit for the BAU. Further conversion of the products SNG and NH_3_ is considered to be out of the work’s scope. The selected functional unit was the total amount of valorizable flue gas produced from natural gas power plants in Germany in 2019, i.e., 1.21·10^11^ kg flue gas year^–1^.^40^ For the BAU, a system expansion approach was used. Notably, we considered the direct emissions from venting the flue gas plus the impact of the BAU (fossil analogs) associated with the equivalent amount of natural gas and NH_3_ produced from flue gas valorization. An attributional approach was selected. We assumed that the O_2_ byproduct from electrolysis was vented, since the market would not be able to absorb this chemical in such quantities.^16^

In the second LCA phase, life cycle inventories (LCIs) are modeled. The foreground system includes all the subprocesses depicted in Figure 1, simulated with Aspen HYSYS v11 and ACM v11. At the same time, the underlying energy and raw material suppliers belong to the background system, here modeled with Ecoinvent v3.5^41^ and accessed through SimaPro v9.2.^42^ All of the inventories, wherever possible, were regionalized for the German or European (RER) region. Specifically, Germany was selected as the reference market, since the country has an energy sector strongly dependent on natural gas imported from abroad and has already in place plans to drastically reduce its reliance on fossil fuels in the upcoming years.^43^ The H_2_ inventory was obtained by combining results from ACM with literature data, assuming H_2_ storage in salt caverns.^16^ Additional details about the inventories can be found in Section 3 in the Supporting Information.

In the third phase, we used the characterization factors proposed by Ryberg et al.^44^ to assess the impact on the control variables of the PBs. Nine Earth-system processes characterized by 11 control variables were considered, i.e., climate change, stratospheric ozone depletion, ocean acidification, biogeochemical flows of nitrogen and phosphorus, land system change, freshwater use, biosphere integrity, atmospheric aerosol loading, and novel entities. The last two of them, atmospheric aerosol loading and novel entities, were omitted from this analysis due to the lack of suitable methods to quantify them. Among all of the PBs, climate change and biosphere integrity are considered as core PBs and, thus, deserve special attention. Nevertheless, since any of the PBs, if trespassed, could lead to catastrophic events, their joint ensemble defines the SOS for human anthropogenic activities.

The impact of each scenario on the Earth’s system was first determined. Considering the set *B* of Earth-system processes and the set *S* of scenarios, the environmental impact of each scenario *s* in each Earth-system *b* was calculated according to eq 1:
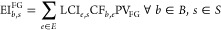
1where LCI*_e,s_* represents the elementary flow *e* linked to the valorization of 1 kg of flue gas in scenario *s*. Note that the values of LCI*_e,s_* are obtained in the second LCA phase (inventory analysis). The parameter CF*_b,e_* denotes the characterization factor quantifying the impact of elementary flow *e* on Earth-system process *b*. These characterization factors were taken from Ryberg et al.^44^ for all the Earth-system processes except for the change in the biosphere integrity, for which the characterization factors proposed by Galán-Martín et al.^25^ derived from ref. (45) were employed instead. Finally, PV_FG_ denotes the amount of flue gas produced from natural gas power plants in Germany in 2019. We next computed the level of transgression (LT) of each scenario with respect to the SOS. The SOS, which denotes the maximum perturbation that the Earth-system processes can tolerate without compromising their long-term stability, is calculated as the difference between the value of the PBs and the natural background levels. Various sharing principles have been proposed to allocate the full SOS for each planetary boundary  among anthropogenic activities,^46^ but there is no consensus yet on which should be universally applied. In this study, we applied a nonegalitarian downscaling to the German gross domestic product in 2019.^47^ The downscaled SOS () is given by eq 2:
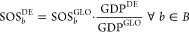
2 where GDP^GLO^ and GDP^DE^ are the global and German gross domestic products in the same reference year, respectively.^47^ Such an approach was selected to reflect both the large impact of the German economy on the European area and beyond, and the direct and indirect implications of a change in the energy sector on the overall economic system of the country. This approach was chosen as the main one presented in this work, since it is the for the BAU. In addition to this approach, an alternative egalitarian downscaling was applied, based on the population, as given in eq 3 (results reported in Figures S11–S13):
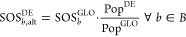
3 where  is the downscaled SOS using the second approach, while Pop^GLO^ and Pop^DE^ are the global and German populations in 2019, respectively.^48^

Hence, we estimated the LT of each scenario () using eq 4:
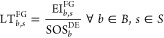
4 where the environmental impact associated with flue gas valorization  is divided by the downscaled SOS of each Earth-system process *b*. When downscaling, a value of  below 100% implies that the scenario does not exceed its ecological budget and, therefore, could be considered sustainable. Conversely, if  is greater than 100%, then the scenario is unsustainable. Exceeding the ecological budget for at least one of the Earth-system processes implies that the scenario is unsustainable in absolute terms since the transgression of one single environmental limit can challenge the resilience of the Earth system. However, since here we are considering the SOS defined for a specific country, high values of the LT below 100% do not imply that the technology is sustainable, because it would leave little room for the others to operate within the SOS too. We also applied the ReCiPe 2016 method (endpoint level, hierarchist approach) to estimate the human health impacts, measured in disability-adjusted life years (DALYs), which represent the years of healthy life lost.^49^ Notably, the resources consumed and the pollutants emitted in our scenarios are linked to water use, global warming, fine particulate matter formation, tropospheric ozone formation, stratospheric ozone depletion, ionizing radiation, and carcinogenic and noncarcinogenic toxicity, which increase the incidence of certain health risks (e.g., undernutrition, respiratory disease, and cancer), damaging human health.

Finally, in step 4 of the LCA methodology, the results are interpreted, and potential recommendations are drawn. Here, we analyzed the impacts of the indicators exceeding the SOS to identify the main hotspots and performed sensitivity analysis on the most relevant parameters involved in the modeling. For the sensitivity analysis, additional details are provided in Section 5 in the Supporting Information.

### Economic Assessment

The routes were compared in terms of the total production cost to valorize one tonne of flue gas, calculated as the summation of the operating expenditure (OPEX) and capital expenditure (CAPEX). The OPEX term accounts for raw materials, utilities, labor, maintenance, property taxation, insurance, and land rent. The CAPEX term was estimated from the equipment cost, computed from the sizes of the process units provided by Aspen HYSYS, and the correlations and installation factors available in SinnottTowler and Sinnott,^50^ except for a few special units such as electrolyzers and membranes, which were costed using other methodologies, as described more in detail in Section 4 in the Supporting Information. A sensitivity analysis of the most relevant parameters was performed according to the criteria described in Section 5 in the Supporting Information.

## Results and Discussion

### Planetary Boundary and Human Health Impact Analysis

**Figure 2:**
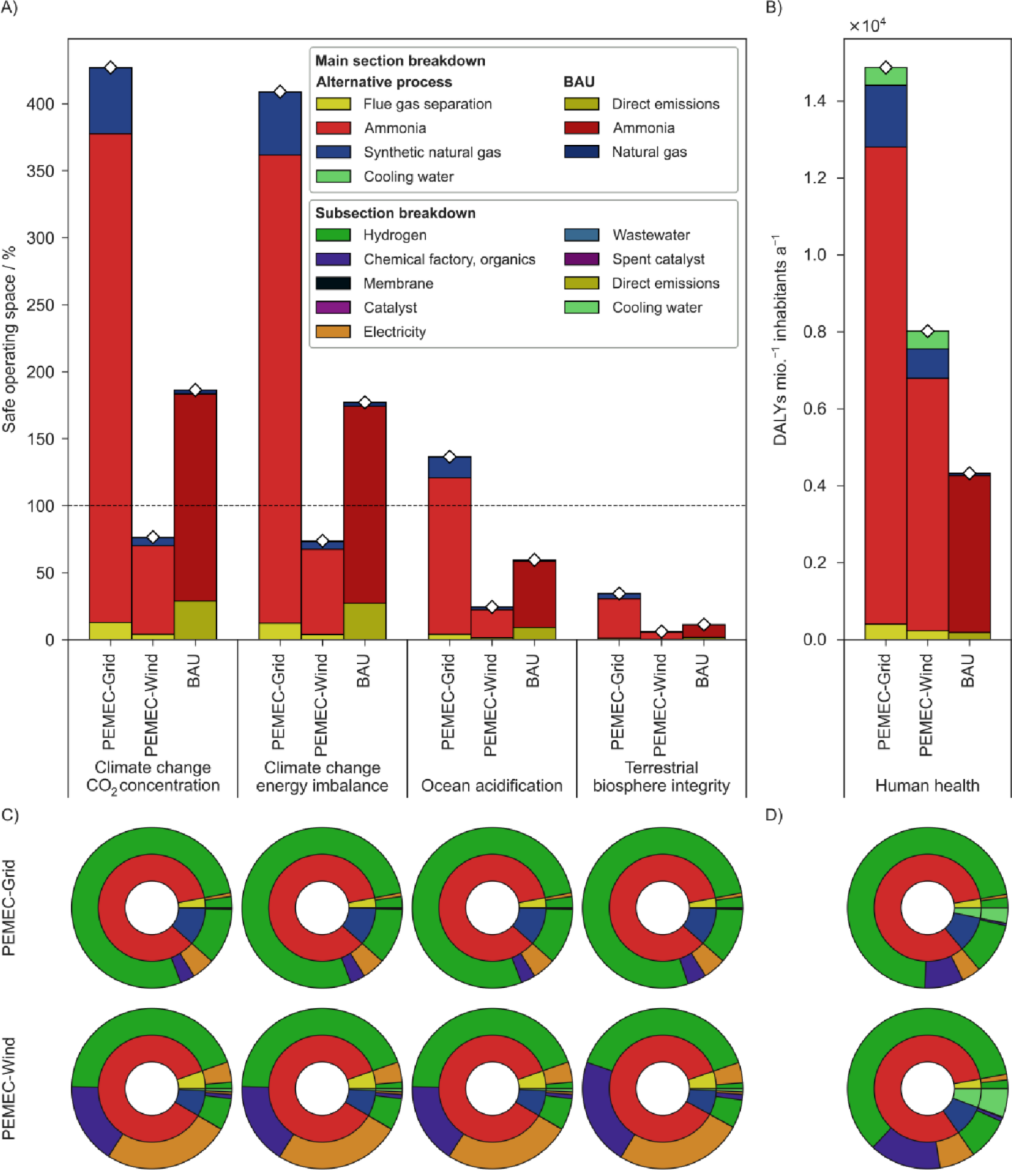
Selection of environmental impact results associated with three scenarios: two flue gas valorization routes using different power sources (PEMEC-Grid and PEMEC-Wind) and the equivalent fossil scenario producing the same quantity of ammonia and natural gas as the alternative scenarios and emitting the flue gas directly into the atmosphere (BAU). The omitted impact categories are reported in Figures S7–S9. (A) Major impacts on the planetary boundaries control variables, quantified in terms of share of the SOS, and breakdown into different plant sections. A nonegalitarian downscaling of the SOS based on the German gross domestic product was used. (B) Results for the endpoint category “human health” of the ReCiPe 2016 methodology (hierarchist approach). (C, D) Detailed subsection breakdown of the impacts depicted in (A) and (B), respectively.

We first focus on the cradle-to-gate impact of the BAU and the PEMEC-based alternative scenarios on the core PBs (climate change and terrestrial biosphere integrity) and an additional PB closely related with climate change, i.e., ocean acidification (Figure 2), using the nonegalitarian downscaling approach. Considering the impacts of the BAU, we observe that its associated LT trespasses the downscaled boundary for the climate change indicators, namely, the CO_2_ concentration and energy imbalance (186.37% and 177.17%, respectively). Despite not trespassing the downscaled threshold, the BAU impact associated with ocean acidification is relevant (59.60%), while the impact on terrestrial biosphere integrity is of minor entity (11.30%). The impact breakdown shows that the contribution associated with producing an amount of NH_3_ equivalent to the one generated by the alternative process dominates the total contribution (82.95–83.35%), followed by the direct emissions stemming from venting the flue gas in the atmosphere (14.18–15.48%). The amount associated with natural gas production in substitution for SNG production in the alternative process has only a marginal impact (15.72–2.47%). The relevant emissions associated with venting the flue gas reflect on their own the large stress exerted on the downscaled climate change Earth system by the sole operation of the natural gas power plants, consisting of 30% of the SOS (ca. 1.68·10^10^ kg CO_2_ year^–1^) even without accounting for the NH_3_ and SNG production associated with the expanded functional unit. Focusing on the human health impacts, the BAU shows impacts of around 4.33·10^3^ DALYs per million inhabitants, with a large dominance of NH_3_ production over the other contributors.

Moving to the flue gas valorization scenarios, all the grid-powered scenarios show an overall impact higher than the BAU in the selected indicators by a factor of 2.29–3.05, with climate change being at the lower end and terrestrial biosphere integrity and human health at the higher end of the range. Specifically, the impacts of such scenarios on climate change and ocean acidification are exceeding the downscaled SOS (427.06, 409.02, and 136.57% for CO_2_ concentration, energy imbalance, and ocean acidification, respectively), making them unsustainable in absolute terms. NH_3_ production holds the largest share (83.37–85.51%), followed by the SNG section (10.80–11.45%). The membrane separation section has negligible impacts (2.72–3.02%). Such stark predominance of the NH_3_ production section is directly related with the N_2_/CO_2_ mass ratio of about 80:15 in the original flue gas stream. When we move to the impacts associated with the wind-based alternative scenario, a different trend can be observed, with impacts being about half of the BAU (0.45–0.58 times the BAU) in the selected control variables. However, the impacts associated with the human health category provide a different picture, with values that are 46.06% lower than the grid-based case but 85.46% higher than the BAU scenario. Once more, the contribution of NH_3_ production dominates over the other sections (81.76–86.75%). Focusing on the control variable climate change – CO_2_ concentration, a further disaggregated breakdown (Figure 2C) reveals the high contribution from electrolytic H_2_ to the overall impacts. The latter is strongly affected by the impact embodied in electricity, resulting in more than 90% of the total impact in the grid-based scenario and more than 50% in the wind-based case. A similar relative contribution of electricity is also reflected in the impacts in ocean acidification and terrestrial biosphere integrity as well. For human health (Figure 2D), such H_2_ contributions represent 84.12% and 70.56% of the total impact for grid- and wind-powered cases, respectively. Considering the different H_2_ requirements of the separate sections (0.171 kg of H_2_ kg^–1^ flue gas for the NH_3_ section and 0.025 kg of H_2_ kg^–1^ flue gas for the SNG production part), the allocation of impacts to different parts of the process is justified. The second largest contributor for the valorization scenarios is the electricity required for the rest of the plant, which corresponds to 4.58–5.42% of the total impact for the grid-based scenario and 8.49–30.21% of the impact for the wind-based case across the indicators here reported.

**Figure 3:**
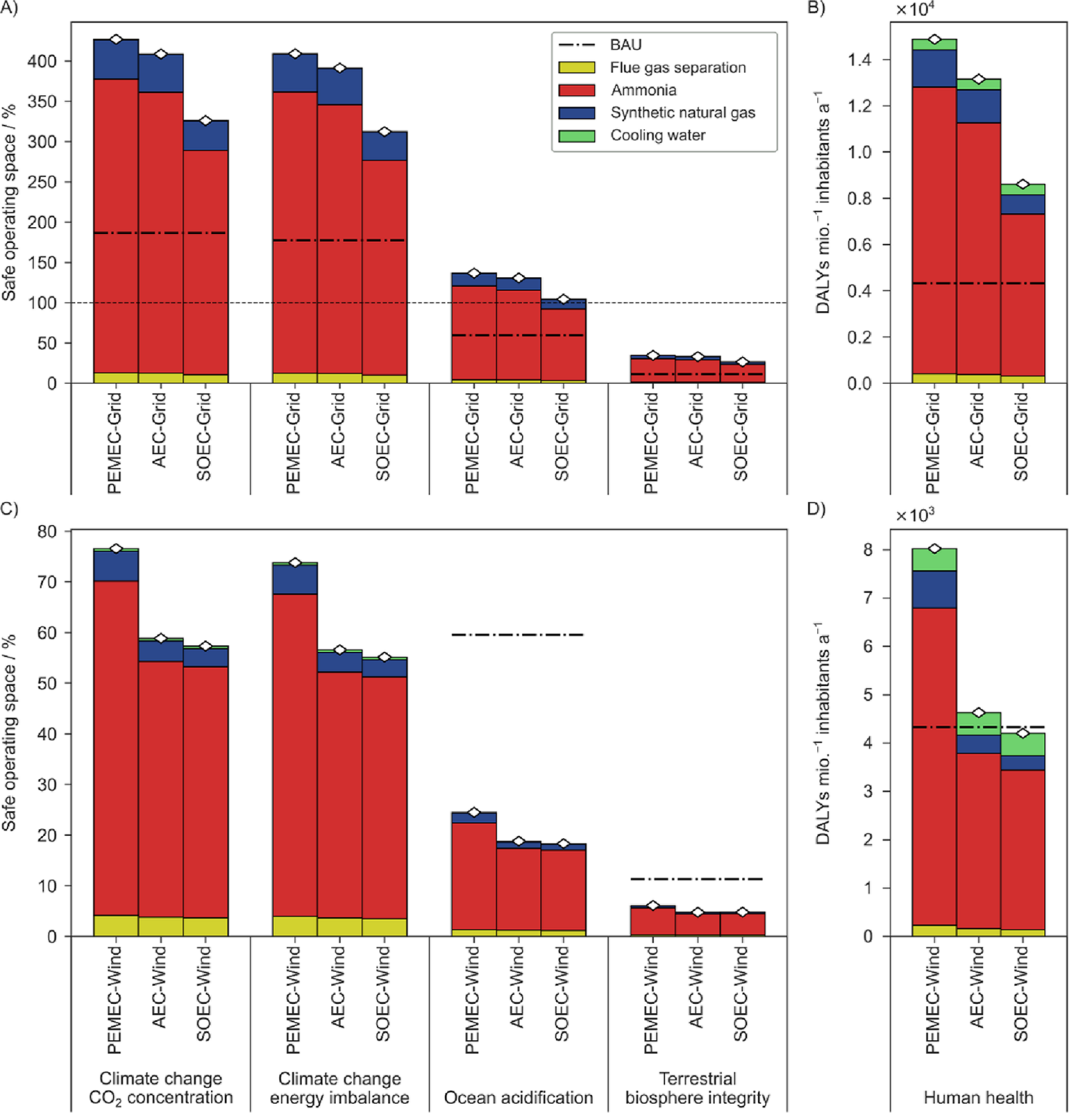
(A,B). Comparison of impacts of alternative configurations for flue gas valorization scenarios adopting grid electricity powering different water electrolytic cell types: PEMEC, AEC, and SOEC. The legend structure is the same as in Figure 2, with the only difference being the BAU, whose total is here reported as a dash-dotted line. (C,D) Comparison of impacts of the same scenarios as (A) and (B) but adopting wind power for the electrolysis section. The selected impact categories and the downscaling method are consistent with the previous figure. A further breakdown of the impacts can be found in Figure S6.

Given the relevance of H_2_ production in the overall environmental performance of the considered flue gas valorization scenarios, different electrolyzer technology types were evaluated (Figure 3). Focusing on the scenarios powered by grid electricity, no alternative scenario outperforms the BAU. According to the ranking for climate change related impacts, PEMEC emerges as the least performing scenario, followed by AEC and SOEC, which manage to achieve a reduction of impacts of 4.29–23.34% and 21.63–25.26% compared to the PEMEC case, respectively. Such a trend results from the energy efficiency of the different technologies, since SOEC has a considerably lower electricity consumption than the alternatives, thanks to the lower overpotential present at higher temperatures (48.9, 47.8, and 36.7 kWh kg^–1^ H_2_ for the PEMEC, AEC, and SOEC, respectively, excluding the rest of the plant). Such a qualitative trend is similar also in the case of ocean acidification and terrestrial biosphere integrity. In both climate change and ocean acidification, all of the scenarios exceed the threshold associated with the downscaled SOS. A trend similar to the said PBs can be observed also in the case of the human health category, with the SOEC-based case performing 42.13% better than the PEMEC-based scenario and AEC showing a performance higher than the PEMEC case by 11.59%.

When moving to the decarbonized scenarios, the ranking for the selected control variables changes, with the BAU being the least performing scenario, followed by the cases based respectively on PEMEC, AEC, and SOEC. This last scenario is, thus, the most sustainable one, with an impact of 57.31% in the control variable with the worst performance, i.e., climate change – CO_2_ concentration. However, in contrast to what is observed when comparing the grid-based scenarios, here the AEC-based case has an almost negligible gap with respect to the best performing scenario, with a relative increase of 2.57–10.15% with respect to the lowest value. This is because of the higher contribution that the construction of the electrolyzer, and specifically the air electrode, plays in carbon-related categories for PEMEC when compared to the other cases (see also Figure S10).^51^

In terms of distributions of the impacts among the different plant sections, the trend is qualitatively similar for all of the scenarios, with the NH_3_ section being associated with most of the impacts for both AEC (78.36–85.80%) and SOEC (78.59–86.57%), followed in most of the cases by the SNG section (6.90–11.49% for AEC and 6.20–11.19% for SOEC). Diving further into the breakdown of the impacts (Figure S6), we find that while for AEC- and PEMEC-based scenarios, H_2_ is still the main contributor (i.e., 40.73–92.05% for AEC and 46.22–91.37% for PEMEC for the considered PBs), the SOEC-Wind scenario sees its predominant share stemming from the grid electricity powering the other sections of the plant. The latter leads to a contribution of 38.72–40.37% against 31.37–35.57% associated instead with H_2_. When focusing on human health impacts, all the grid-based scenarios perform worse than the BAU by 191.56–243.82%, with PEMEC being the worst performing and SOEC the best. However, when shifting to renewable-based H_2_ production, a qualitatively different trend is observed for the SOEC-based case with respect to the PEMEC- and AEC-based scenarios. In fact, while the use of PEMEC and AEC still results in a higher human health footprint than the BAU (+85.46 and +70.16%, respectively), SOEC can perform 2.85% better than the industrial baseline. This is thanks to the drastically different impact associated with the electrolyzer construction, which is in line with previous studies used here as a source for the LCIs (see also Figure S10).^51^ The high impact of PEMEC electrolyzer construction in human health stands out particularly because of the high contribution in fine particulate matter formation due to the environmental burden of platinum group metal extraction. Also in the case of AEC, the majority of the human health impacts derive from the high contribution in the same category, which is associated in this case with the nickel requirements, much higher than in the case of SOEC.

The remaining assessed PBs are presented in Figures S7 and S8. Freshwater use is the PB showing the highest gap between flue gas valorization scenarios and BAU in favor of the latter, with valorization scenarios occupying a share of SOS up to 11.76%, while the BAU share stays below 0.1%. Such results are partly due to the notably high evaporation rate assumed for the cooling water of about 38%. However, it should be stressed that, even assuming near-to-zero cooling water evaporation rates, the freshwater use impacts associated with the valorization scenarios would still be more than 1 order of magnitude higher than in the BAU, highlighting the relatively low contribution that the electricity source plays for such an indicator. The overall ranking for this PB sees PEMEC performing worse than AEC, with SOEC performing best among the proposed alternative scenarios, and grid-powered scenarios performing worse than the wind-powered ones. A similar ranking, where PEMEC-Grid has the highest impacts and the BAU the lowest, can be found also for the two indicators associated with biogeochemical flows, whose worst-case scenario takes 4.66% and 0.14% of the SOS for nitrogen and phosphorus flows, respectively. Regarding stratospheric ozone depletion, a trend qualitatively equivalent to the human health impacts category can be highlighted, with PEMEC-Grid, the worst performing scenario, taking 0.82% of the associated SOS. Such a qualitative resemblance is due to the influence of stratospheric ozone depletion causing skin cancers, thus directly affecting human health. Finally, in terms of level of transgression of the land-system change PB, all the scenarios take less than 0.01% of the SOS associated with this control variable. In this case, the BAU scenario has higher impacts than the other cases, while SOEC-Wind is the best performing scenario, with an impact even below 0.001% of the SOS.

If an alternative egalitarian downscaling approach based on population is applied (Figures S11–S13), the performance of all of the scenarios worsens substantially. Specifically, the impacts associated with climate change indicators trespass the SOS for all of the scenarios (227.00–1757.71%), with the BAU trespassing the boundary even when accounting for the sole contribution associated with the direct emissions (see Figure S11). Moreover, the BAU and the grid-based valorization scenarios exceed the SOS also for ocean acidification control variable (up to 562.10%). The grid-based alternative scenarios also exceed the biosphere integrity boundary (up to 142.04% of the SOS). Other control variables do not see any of the assessed scenarios exceeding the downscaled SOS.

Furthermore, focusing on other impacts associated with other ReCiPe 2016 endpoint categories (Figure S9), the qualitative ranking for the damage on the ecosystems category shows that the BAU performs worse than AEC-Wind and SOEC-Wind but better than PEMEC-Wind. As for the previously discussed indicators, PEMEC always performs worse than the other H_2_ routes, and SOEC always performs better than the alternatives. Finally, the BAU is the worst performing scenario in terms of depletion of resources, with all the grid-based and wind-based valorization scenarios having an impact of about half as much if not less than the fossil standard.

The parameters most relevantly associated with the highlighted hotspots for the valorization scenarios, namely, the electrolyzer energy consumption and the electrolyzer size, affecting linearly the overall system electricity consumption and the electrolyzer construction size, were varied in a sensitivity analysis (see Figure S16 and additional details in Section 5 in the Supporting Information). Such analysis highlighted how the parameter affecting the most the results is the electrolyzer energy consumption, with values of up to 0.92 for the ratio of relative change in the output versus a corresponding relative change in this input parameter. The impact category experiencing the widest range of variability is human health (up to −24.8/+23.3% variation with respect to the base case), while, in terms of control variables, stratospheric ozone depletion has the highest variability (−17.4/+16.1%).

### Economic Assessment

**Figure 4:**
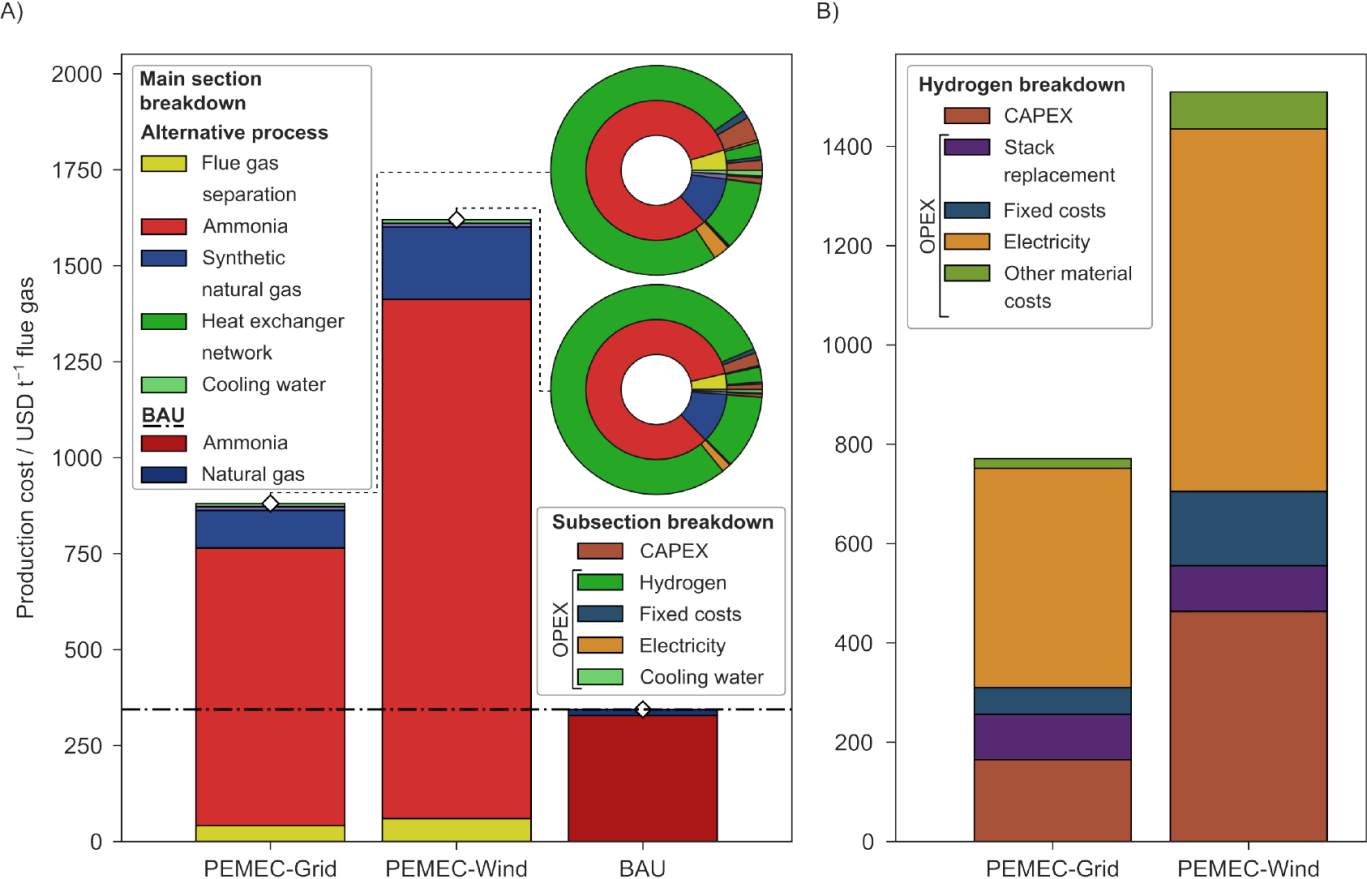
(A) Economic impacts associated with the three scenarios: PEMEC-Grid, PEMEC-Wind, and BAU. The breakdown into different plant sections is displayed both in the bar and in the pie charts, and a detailed subsection breakdown is presented in the latter chart type as well. The total for the BAU is also visible as a dash-dotted horizontal line. (B) Breakdown of hydrogen production cost for the two electrolysis-based scenarios: PEMEC-Grid and PEMEC-Wind. The other scenarios for both subfigures are reported in Figures S14 and S15.

When moving to the economic performance of the assessed scenarios (Figure 4A), the ranking changes sensibly, with the BAU corresponding to the lowest costs (344 USD tonne^–1^ flue gas) and the PEMEC-Wind to the highest (1620 USD tonne^–1^ flue gas). For the BAU, almost the entirety of the costs are associated with the NH_3_ production (95.41%), with natural gas covering the remaining share. A partially similar trend can be found for the valorization scenarios, with the NH_3_ plant section covering 82.14–83.51% of the total costs for PEMEC-based scenarios followed by the SNG section, with 11.11–11.63% of the total cost share. The membrane separation section takes a relatively small share of the total (3.70–4.71%). Looking further into the breakdown of the valorization scenarios, H_2_ emerges again as the main contributor, taking 87.62–93.21% of the share, followed by CAPEX (3.48–6.40%) and electricity (1.64–3.01%). When looking into the costs associated with H_2_ production (Figure 4B), electricity takes the largest cost share (48.38–57.28%), followed by the CAPEX and the stack replacement costs, which together account for 33.23–36.78% of the total H_2_ cost. When moving to other electrolyzer types (Figures S14 and S15), the trend shows SOEC as the least cost-performing technology when powered by grid electricity (901 USD tonne^–1^ flue gas), followed by PEMEC (881 USD tonne^–1^ flue gas) and AEC being the most cost-effective valorization route (820 USD tonne^–1^ flue gas). At the same time, in the case of wind-based scenarios, AEC still leads the ranking of valorization pathways, but PEMEC performs slightly worse than SOEC (+1.26%), stressing once more how the combination of different energy efficiencies and electricity types can constitute a major discriminant between scenario performances. Furthermore, such trends reflect the level of maturity of the technologies, since AEC is already considered in mature commercialization, while PEMEC and SOEC are at the incipient or early commercialization stage. The proposed alternative processes in the decarbonized form would need a CO_2_ taxation between 843 and 1134 USD tonne^–1^ CO_2_ to reach breakeven with the BAU.

A sensitivity analysis is presented in Figure S17, focusing on four key parameters: electrolyzer energy consumption, electrolyzer size, electrolyzer cost, and levelized cost of electricity from wind fed to the electrolyzer. All of the ratios of the relative output variation versus the relative input variation are within the range 0.29–0.56, with no clear trend that can be highlighted across different scenarios. The total cost variation lies within a range of about −71.5/+125.4% with respect to the base case across the assessed valorization scenarios. Notably, for the lower bound, this would imply a cost decrease of 68.3, 67.0, and 71.5% for the PEMEC-Wind, AEC-Wind, and SOEC-Wind scenarios, respectively, driving down the corresponding cost gap with the BAU to only 49.1, 39.1, and 35.7%.

## Conclusions

The present work assessed an integrated process valorizing flue gas from natural gas power plants into NH_3_ and SNG, with the help of electrolytic H_2_ produced through PEMEC, AEC, and SOEC. The absolute environmental impact on Earth’s natural limits was quantified with the help of the Planetary Boundaries framework downscaled to the German market and complemented with the assessment of human health impacts. First, it was found that the current BAU, calculated using expanded system boundaries, trespasses the SOS associated with climate change and corresponds to high shares of the SOS also in ocean acidification and terrestrial biosphere integrity. Since the main proposed downscaling approach was the most optimiztic, it can be inferred that such a process cannot be considered sustainable in the climate change control variables using any other approach that restricts the environmental budget further. On one hand, a flue gas valorization system could substantially reduce impacts in climate change and terrestrial biosphere integrity, more so when using decarbonized H_2_ generated with a solid oxide electrolyzer. In such a case, the pressure on the currently most exerted boundaries, i.e., climate change, ocean acidification, and biosphere integrity, might be significantly reduced. On the other hand, human health impacts might worsen in the integrated system depending on the power source and electrolyzer type, particularly when a proton exchange membrane and alkaline electrolyzers are used. Furthermore, burden shifting was observed in freshwater use and biogeochemical flows for all the assessed alternative scenarios. When adopting a different downscaling principle for the planetary boundaries framework, such as an egalitarian population-based approach, all of the impacts worsen. However, the use of different downscaling principles does not affect the relative sustainability ranking of the assessed scenarios, and the associated conclusions remain the same.

In addition to this, a technoeconomic assessment showed that the investigated flue gas valorization scenarios cannot compete with the BAU, at the current state and without further valorization of side products or process integration with other systems. This yields costs up to four times larger than in the industrial standard, considering a H_2_ price of 3.55–7.57 USD kg^–1^ H_2_ when wind-energy is used. Such costs translate into a carbon abatement cost of 843–1134 USD tonne^–1^ CO_2._ This range is above some negative emission technology costs of biomass gasification with Fischer–Tropsch fuel synthesis with carbon capture and storage (CCS) (about 375–534 USD tonne^–1^ CO_2_) or biogas production from biomass anaerobic digestion with CCS(ca. 239–568 USD tonne^–1^ CO_2_).^52^ Notably, our study considers prepandemic cost values for the BAU, thus failing to capture how fossil fuel prices might increase due to disruptions in the natural gas supply chain, as in Europe in 2022. Indeed, recent studies pointed out how, in such cases, renewable-based chemicals might become economically competitive.^53,54^ Furthermore, future projections for similar power-to-SNG routes proved that such technologies might become cost-competitive with the BAU, considering a substantial decline in the cost of electricity and of the electrolyzer, improvements in the electrolyzer efficiency, and the valorization of byproducts such as oxygen.^13−15^

Proton exchange membrane and solid oxide electrolyzers are still in the incipient or early commercialization stage. Hence, the mentioned improvements might contribute to narrowing the gap between standard technologies and emerging low-carbon ones while leveraging intermittent power that would otherwise be curtailed.

## Data Availability

The data underlying this study are openly available in Zenodo under the DOI: 10.5281/zenodo.10048201. Additional data underlying this study are available from the corresponding author upon reasonable request.

## Supporting Information Available

Modeling details for the processes modeled in Aspen HYSYS and Aspen Custom Modeler; life cycle inventories; additional details on planetary boundaries methodology, economic parameters used to calculate hydrogen cost and other OPEX, and CAPEX calculations; further details on sensitivity analysis, environmental assessment metrics, and economic assessment metrics; and results of the sensitivity analysis (DOCX)
