# Supplementary material for: Absolute Sustainability Assessment of Flue Gas Valorization to Ammonia and Synthetic Natural Gas
Source: ACS Sustain Chem Eng. 2023 Dec 8;11(50):17718–27. doi: 10.1021/acssuschemeng.3c05246 (PMC10731640; doi:10.1021/acssuschemeng.3c05246)
Supplement: Supplementary file 1 — sc3c05246_si_001.docx [file sc3c05246_si_001.docx]

Supporting Information

Absolute Sustainability Assessment of Flue Gas Valorization to Ammonia and Synthetic Natural Gas

Sebastiano Carlo D’Angelo,^†^ Julian Mache,^†^ Gonzalo Guillén-Gosálbez^†,*^

^†^Institute for Chemical and Bioengineering, Department of Chemistry and Applied Biosciences, ETH Zurich, Vladimir-Prelog-Weg 1, 8093 Zurich, Switzerland.

^*^Corresponding author: Gonzalo Guillén-Gosálbez: [gonzalo.guillen.gosalbez@chem.ethz.ch](mailto:gonzalo.guillen.gosalbez@chem.ethz.ch)

Number of pages: 52

Number of figures: 17

Number of tables: 18

This document contains the supporting material of the main article, which is organized as follows. **Section 1** describes the scenarios considered in this work. **Section 2** describes the process flowsheets developed for the analysis. **Section 3** provides further information on the environmental assessment, reporting the assumptions and the data sources associated with the life cycle inventories (LCIs). **Section 4** provides further details on the economic assessment, while **Section 5** provides further details on the sensitivity analysis. Finally, **Section 6** includes additional results: (*i*) the impacts for the single planetary boundaries (PBs) that are not described in the main manuscript with their breakdown, for the non-egalitarian downscaling approach, (*ii*) all the impacts and the contributions for each endpoint category of the ReCiPe 2016 methodology, (*iii*) all the impacts and the contributions for all the PBs using the alternative egalitarian downscaling approach, (*iv*) the economic analysis for all the investigated scenarios, (*v*) the results of the sensitivity analysis.

1. Definition of scenarios and acronyms

The scenarios considered in the analysis and the corresponding labels are provided in **Table S1**.

**Table S1.** Correspondence between labels and scenarios reported in this study.

| **Label** | **Scenario** |
| --- | --- |
| BAU | Business-as-usual (ammonia, NH_3_, from steam methane reforming, SMR; natural gas from fossil extraction; direct emissions stemming from natural gas power plant, NGPP, and vented without carbon capture). |
| PEMEC-Wind | Hydrogen (H_2_) from proton exchange membrane (PEM) electrolysis, electricity demand for the electrolyzer satisfied by wind energy, electricity demand for the rest of the plant satisfied by the German grid 2020 mix. |
| PEMEC-Grid | H_2_ from PEM electrolysis, electricity demand for the electrolyzer and for the rest of the plant satisfied by the German grid 2020 mix. |
| AEC-Wind | H_2_ from alkaline electrolysis, electricity demand for the electrolyzer satisfied by wind energy, electricity demand for the rest of the plant satisfied by the German grid 2020 mix. |
| AEC-Grid | H_2_ from alkaline electrolysis, electricity demand for the electrolyzer and for the rest of the plant satisfied by the German grid 2020 mix. |
| SOEC-Wind | H_2_ from solid oxide electrolysis, electricity demand for the electrolyzer satisfied by wind energy, electricity demand for the rest of the plant satisfied by the German grid 2020 mix. |
| PEMEC-Wind | H_2_ from solid oxide electrolysis, electricity demand for the electrolyzer and for the rest of the plant satisfied by the German grid 2020 mix. |

1. **Process flowsheets**

Three models were built in Aspen HYSYS^®^ v11, assuming hydrogen (H_2_) production from proton exchange membrane (PEMEC), alkaline (AEC), and solid oxide electrolytic cells (SOEC), respectively. An overview of the underlying common model is given by **Figure S1**. The models are used to provide the mass and energy flows required to quantify the life cycle inventory (LCI) for all the scenarios, except for the business-as-usual (BAU). In addition to this, the heat integration of the processes modeled in Aspen HYSYS^®^ v11 was performed with the embedded tool Aspen Energy Analyzer^®^ v11, which provided the heating and cooling utilities consumption and the heat exchanger network design. After this step, the mass and energy inputs and outputs associated with flue gas valorization are first retrieved from the Aspen model described below (foreground system), and the associated impact is then calculated for every scenario by considering the corresponding electricity source, which varies according to the scenario. The inventories for the BAU are determined by combining data from the literature with data from Ecoinvent v3.5.^1^ The LCIs for other background activities, *i.e.*, nitrogen (N_2_) production, electricity generation, etc., are also retrieved from Ecoinvent, in some cases combining data therein with information from the literature.

| 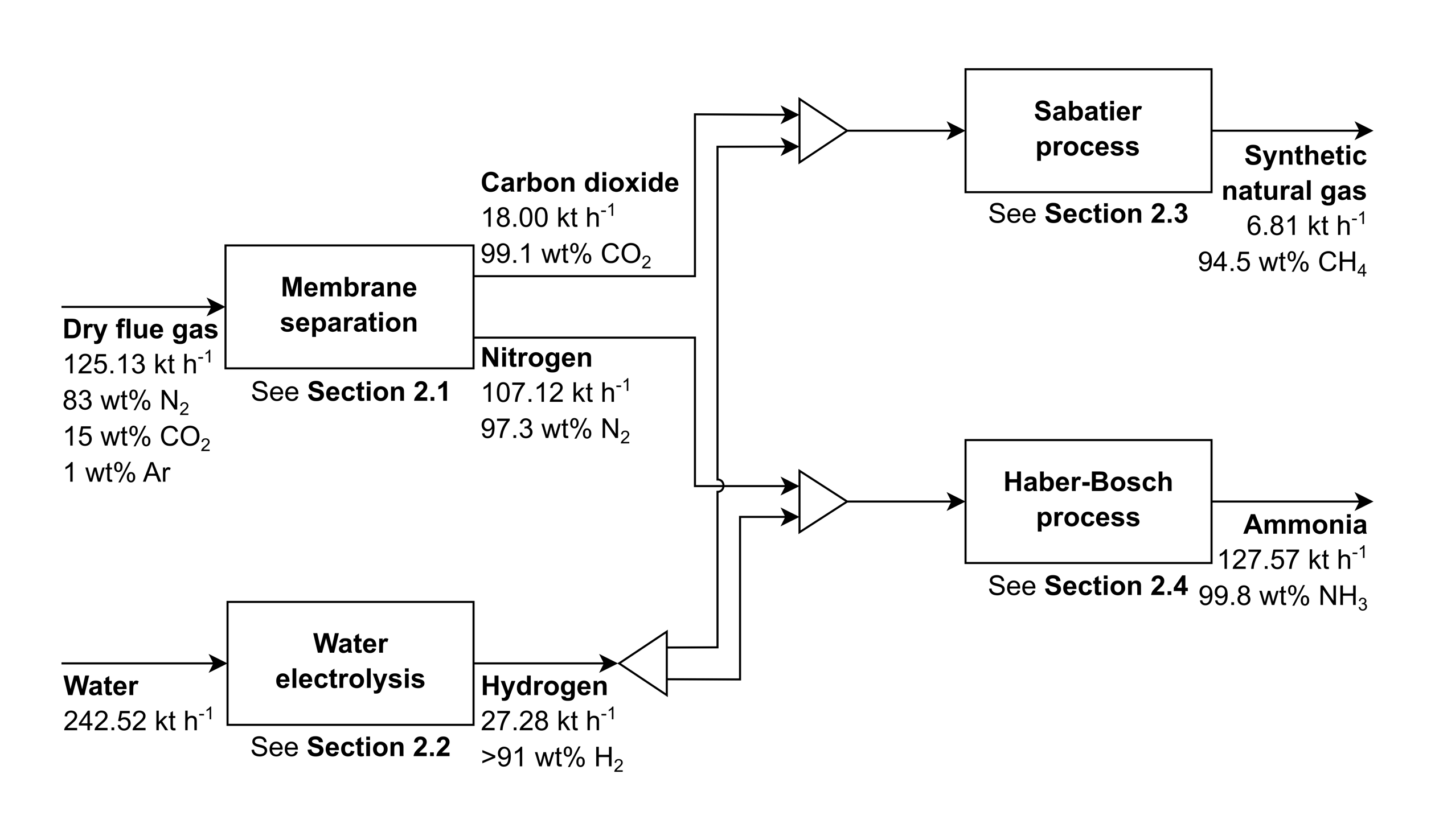 |
| --- |
| **Figure S1.** Flowsheet with the general overview on the process considered in this study. |

- 1. **Flue gas separation**
     1. **Flue gas pretreatment**

**Table S2** provides the flue gas composition assumed for this study. The single plant is designed to treat a given amount of flue gas considering a maximum availability of energy imposed by the use of 10% of the total electricity produced by wind in Germany, which we assume it could be used for electrolytic H_2_ production for the flue gas valorization. Given an annual wind energy production of 131.7 TWh in 2020^2^ and an annual plant operating time of 8000 h, this represents an average consumption of 1.65 GW, corresponding to about 136 kt h^−1^ of wet flue gas.^3^ This quantity is thus considered as reasonable capacity for a single plant, and the overall amount of treated flue gas is later upscaled in the environmental analysis to account for the treatment of the flue gas from all the natural gas power plants in Germany.^4^ Specifically, the flue gas stream was considered to stem from a combined-cycle natural gas power plant with a flue gas recirculation of 0.42. The flue gas recirculation reduces the total flow rate and oxygen (O_2_) concentration of the flue gas while increasing the carbon dioxide (CO_2_) concentration, which is beneficial for the subsequent separation step. As the original source does not consider explicitly the presence of argon (Ar), it is assumed that some of the nitrogen (N_2_) is replaced by argon with a ratio of N_2_ to Ar equal to the one present in the air. The molar concentrations of the other components, namely, water and O_2_, were not modified.^3^ Flue gas is assumed to be fed at 200 °C and 20 bar.

**Table** **S2.** Flue gas composition, modified from ref.^3^

| **Component** | **Molar fraction** | **Mass fraction** |
| --- | --- | --- |
| N_2_ | 0.7965 | 0.7676 |
| CO_2_ | 0.0920 | 0.1393 |
| H_2_O | 0.0670 | 0.0415 |
| O_2_ | 0.0350 | 0.0385 |
| Ar | 0.0095 | 0.0131 |

O_2_ acts as catalyst poison in the Haber-Bosch (HB) process and its concentration therefore needs to be kept below 10 ppm.^5^ The O_2_ concentration in synthetic natural gas (SNG) also needs to be kept below 10 ppm by law in Germany for safety reasons.^6^ Thus, an O_2_ removal step is necessary, and it was selected as first step of the flue gas separation process. This is also advantageous as the membrane model used at a later stage is originally written for a binary mixture and the presence of O_2_ would create additional uncertainty in the model validity. There is not yet a commercially viable O_2_ separation technique able to achieve the desired purity, with cryogenic distillation being very energy-intensive and membrane separation not selective enough. Thus, catalytic removal of O_2_ is applied using H_2_ as a reducing agent.^7^ H_2_ is fed in stoichiometric amounts and reacts as:

| 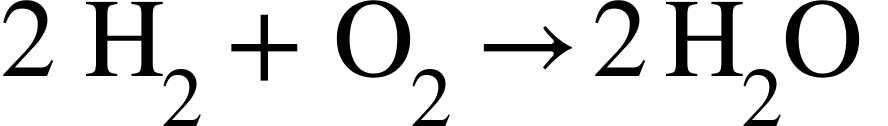 | (Equation S1) |
| --- | --- |

H_2_ might also react with CO_2_ to form CO. However, this is not considered a problem in this application. In fact, as CO is chemically similar to CO_2_, most of it is assumed to accumulate in the CO_2_‑rich stream for methanation. As in the Sabatier reaction it is an intermediate, as described in **Section 2.3**, traces of CO in the feed should not pose any issues. For the feed for the HB process, traces of CO_2_ are removed by methanation as described in **Section 2.4** using the Sabatier reaction as well. The reaction is modeled as an isothermal equilibrium reactor operating at 350 °C.^6^ As the reaction is exothermal, cooling is necessary even though the reactor temperature is higher than the feed temperature. CO formation is not considered, in line with previous assumptions.

To avoid possible issues with water, like condensation in the membrane units, the flue gas is dried before entering the first membrane module. First, the flue gas is cooled to 30 °C and flashed to reduce the water content. After this step, the stream is further dried using triethylene glycol (TEG) dehydration. Thereby, water is removed by passing the flue gas through an absorber with TEG, which absorbs the water in countercurrent. Absorption occurs at the same pressure as the deoxygenation, namely, 20 bar. The water-rich TEG solution is recovered by distillation at ambient pressure.

- - 1. **Membrane model**

The membrane model is modified from the work by Zanco et al..^8^ The model is coded in Aspen Custom Modeler^®^ (ACM) language, setting up the ordinary differential equation (ODE) system on the level of one fiber using dimensionless variables and extending it to a full module.

The following values are first calculated:

| Area per fiber (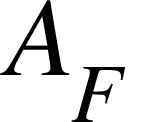) | 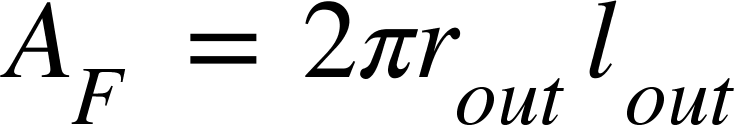 | (Equation S2) |
| --- | --- | --- |
| Flow per fiber (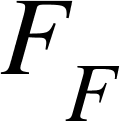) | 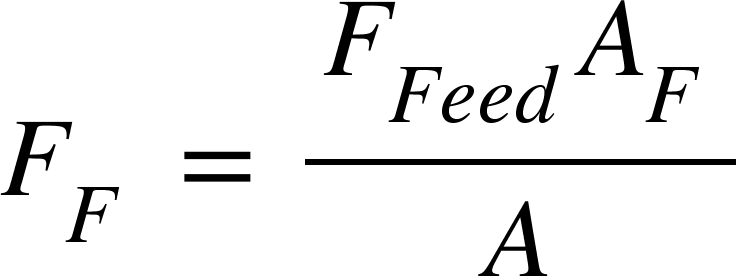 | (Equation S3) |
| Hydraulic radius on permeate side (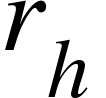)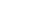 | 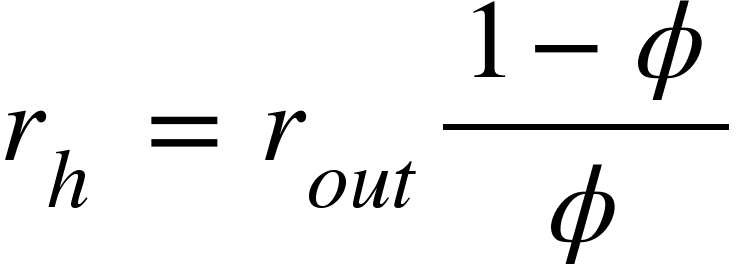 | (Equation S4) |
| Dimensionless membrane area (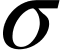) | 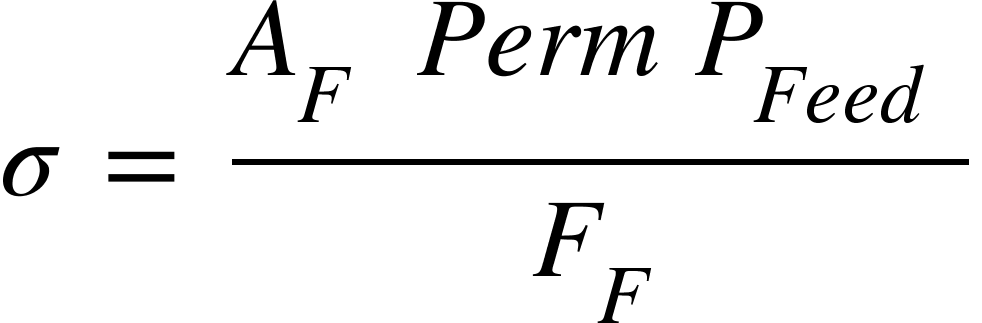 | (Equation S5) |
| Dimensionless pressure drop parameter, retentate (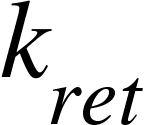) | 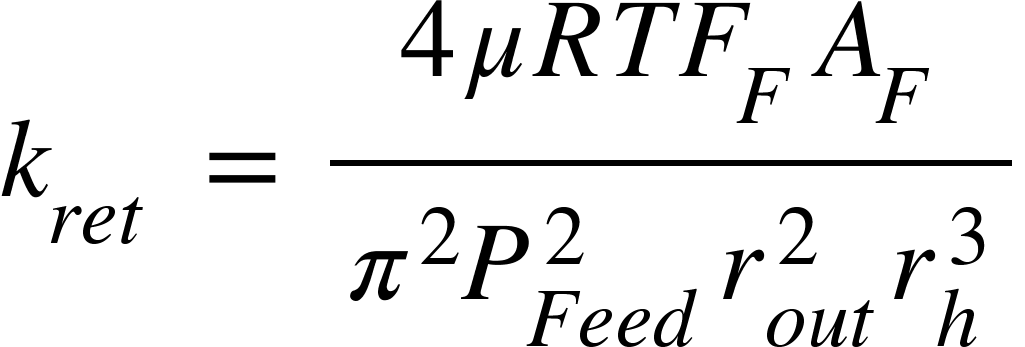 | (Equation S6) |
| Dimensionless pressure drop parameter, permeate (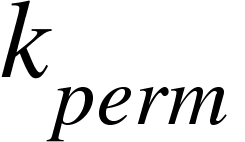) | 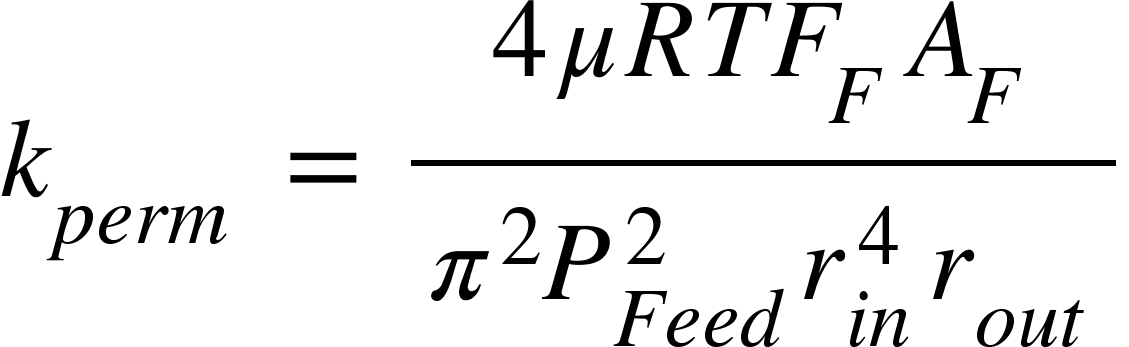 | (Equation S7) |

where:

-
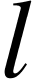
 is the fiber length;
-
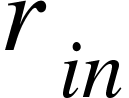
 and
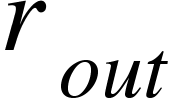
 are the inner and outer fiber radius, respectively;
-
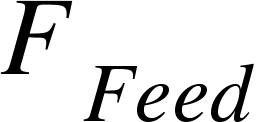
 is the feed flow rate;
-
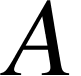
 is the total membrane area;
-
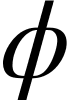
 is the fibers packing ratio;
-
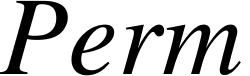
 is the permeance of CO_2_;
-
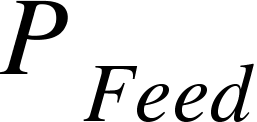
 is the feed pressure;
-
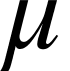
 is the dynamic viscosity of the retentate gas.

The fiber is described by the following ODE system:

| 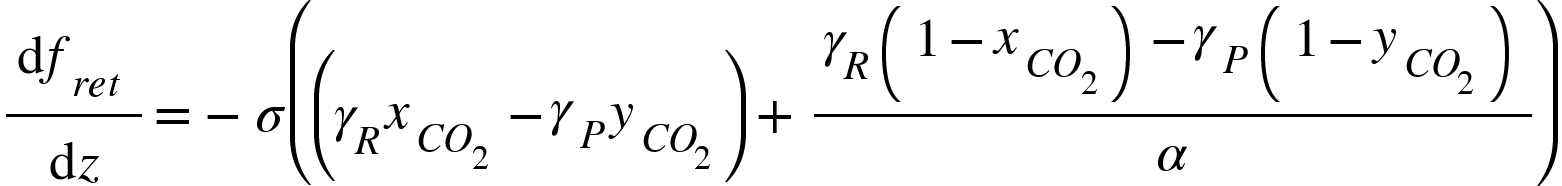 | (Equation S8) |
| --- | --- |
| 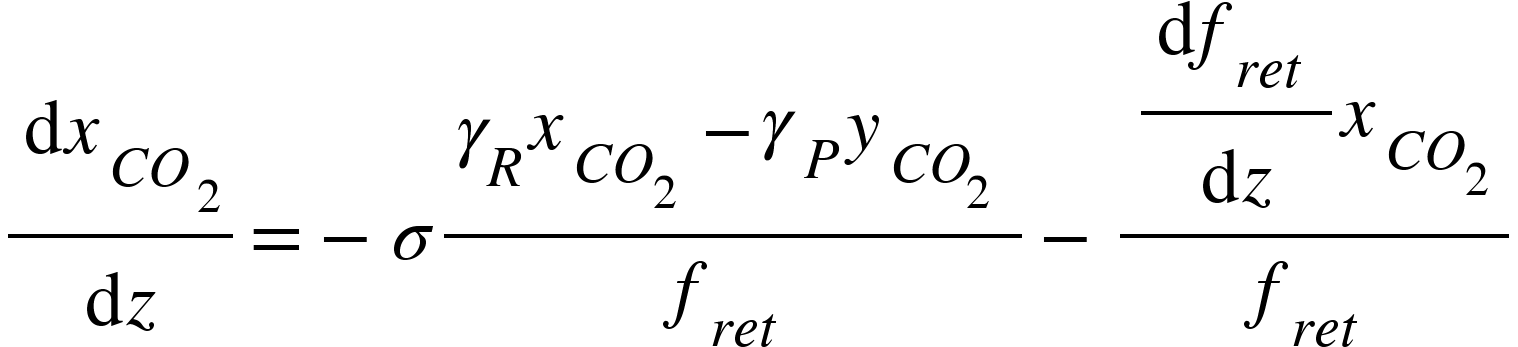 | (Equation S9) |
| 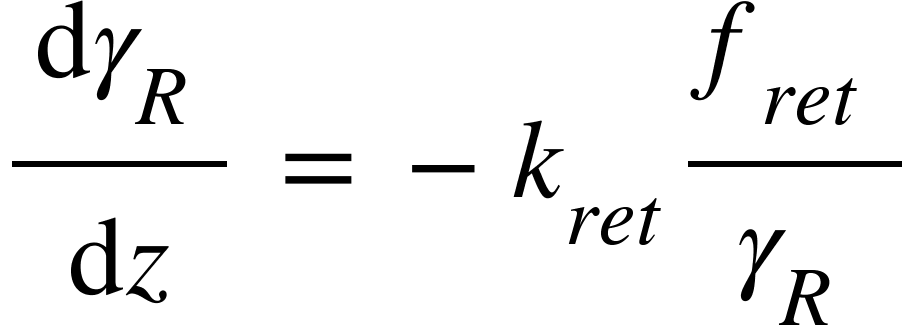 | (Equation S10) |
| 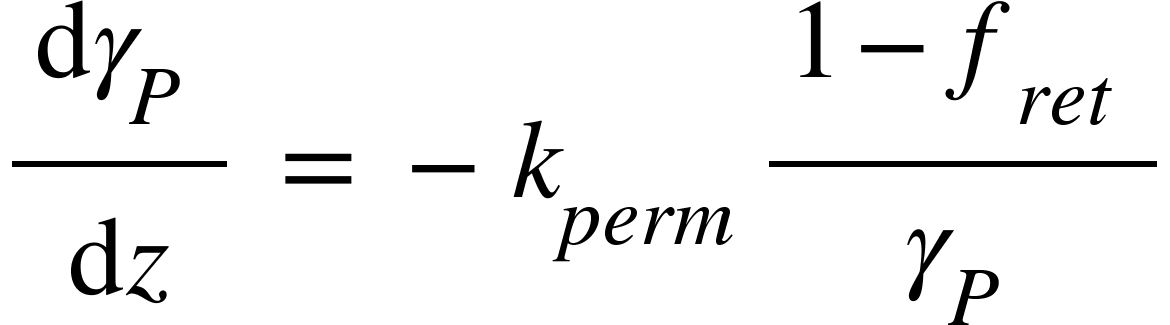 | (Equation S11) |

where:

-
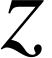
 is the axial coordinate;
-
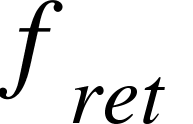
 is the dimensionless retentate flow rate;
-
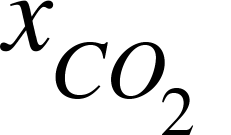
 and
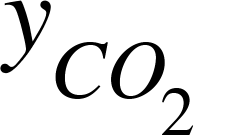
 are the CO_2_ molar fractions on the retentate and the permeate side, respectively;
-
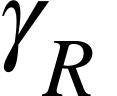
 and
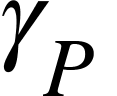
 are the dimensionless pressures on the retentate and permeate side;
-
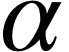
 is the selectivity of the membrane to CO_2_ with respect to N_2_.

The dimensionless flow rates and pressures are defined as a fraction of the feed flows rate and pressure, respectively. The model originally assumes flue gas as a two-component mixture of only CO_2_ and N_2_. It is expanded by assuming the same permeability for Ar as for N_2_, which does not change the equations with respect to CO_2_. The molar fraction
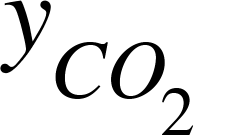
 is calculated as:

| 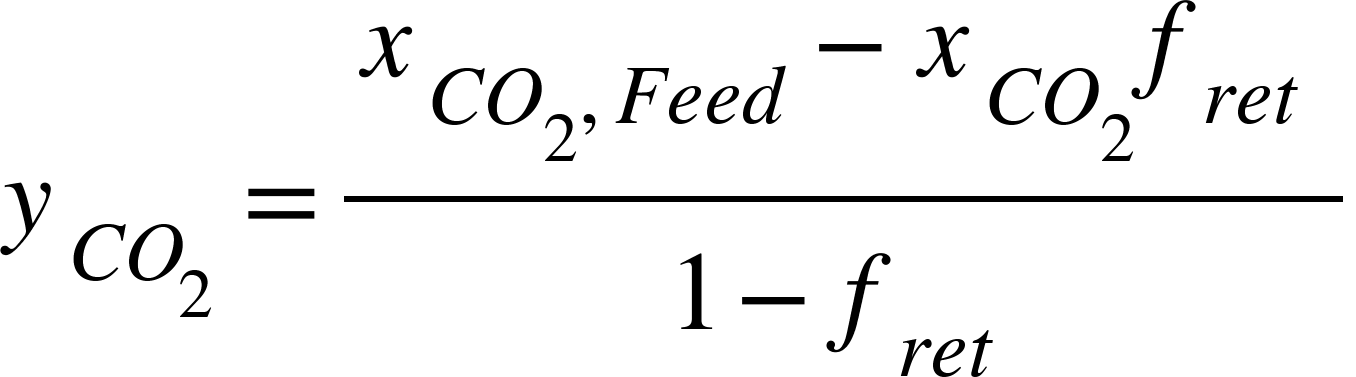 at 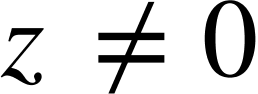 | (Equation S12) |
| --- | --- |
| 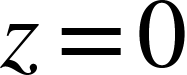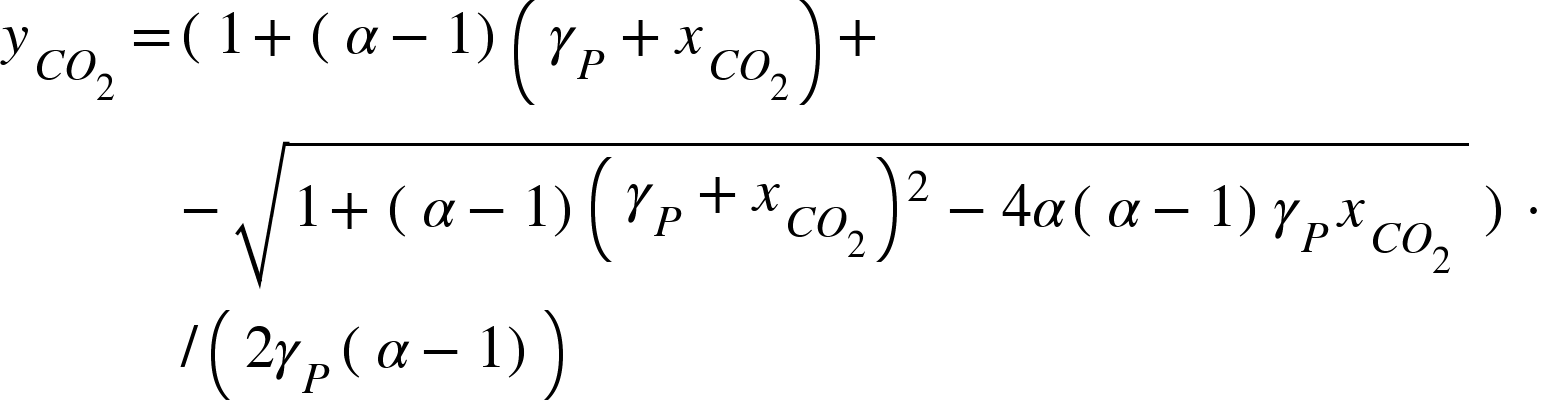 at | (Equation S13) |

with
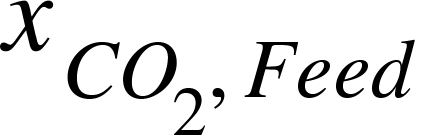
 being the CO_2_ molar fraction of the feed. The provided boundary conditions are the following:

| 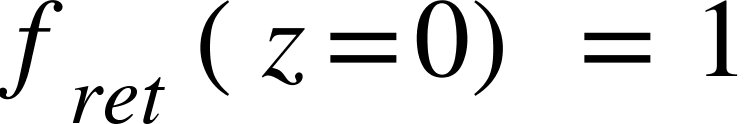 | (Equation S14) |
| --- | --- |
| 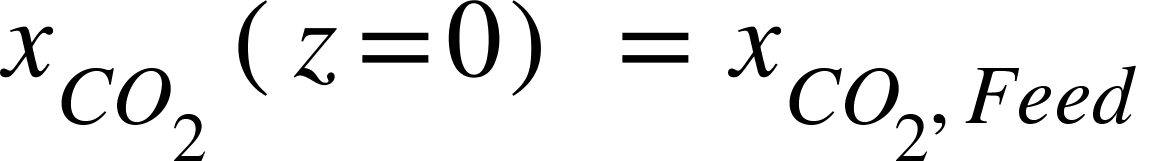 | (Equation S15) |
| 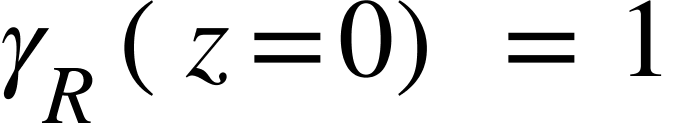 | (Equation S16) |
| 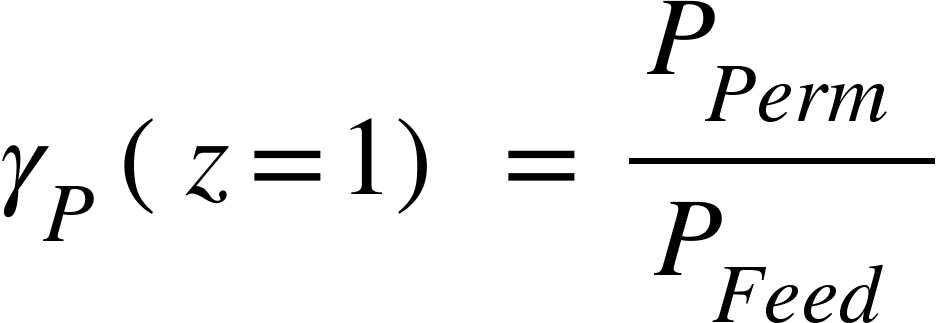 | (Equation S17) |

with the permeate outlet pressure,
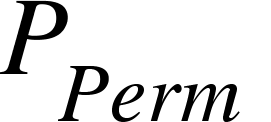
,being one of the specifications of the membrane.

After solving the ODE system, the outlet properties are calculated from the dimensionless quantities on the outlet and simple overall material balances over the components. It is assumed that the ratio of Ar to N_2_ in the outlets is the same as in the feed. The separation is considered isothermal. A membrane able to tolerate pressures up to 20 bar, as for instance an Evonik Sepuran^®^ Green membrane,^9^ is considered, with properties listed in **Table S3** for an assumed effective membrane thickness of 50 nm together with the assumed parameters. For
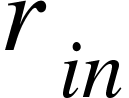
,
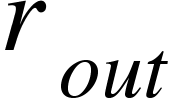
, and
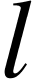
 arbitrary values within the reasonable size range were chosen. As the pressure drop along the membrane is very low, these three values only have a minor effect on the performance of the modules. For
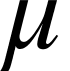
, an average value of the values from the feed obtained from Aspen HYSYS^®^ is used.^8^

**Table** **S3.** Parameters associated with the inlet flow properties and the membrane used for the membrane model.^8^

| **Parameter** | **Value** |
| --- | --- |
| Fiber length (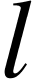) [m] | 1 |
| Inner fiber radius (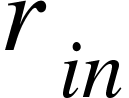) [µm] | 400 |
| Outer fiber radius (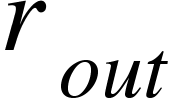) [µm] | 667 |
| Fibers packing ratio (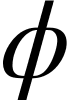) [-] | 0.55 |
| Permeance of CO_2_ (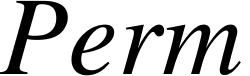) [kmol^−1^ bar^−1^ m^−2^] | 0.02187 |
| Selectivity of the membrane to CO_2_ with respect to N_2_ (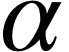) [-] | 30.4 |
| Dynamic viscosity of the retentate gas (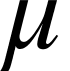) [mPas] | 0.018 |

- - 1. **Membrane module assembly**

In order to achieve the desired purities of over 98% in N_2_ and CO_2_, an assembly of 4 membrane modules was chosen. The corresponding flowsheet is presented in **Figure S2**. The retentate side gets enriched in N_2_ while the permeate side gets enriched in CO_2_. Thus, membrane 1 (M1) serves as a preliminary separation, membrane 2 (M2) serves for further enrichment of the N_2_ stream while membranes 3 (M3) and 4 (M4) serve to further enrich the CO_2_ stream. Due to the significant pressure drop across the membrane, the permeate needs to be recompressed significantly and afterward cooled before being fed to another membrane. The retentate only suffers from a very mild pressure drop after each membrane and only needs recompression if it is recycled back to maintain a driving force in the recycle loop. No cooling is necessary.

The permeate from M2 is slightly enriched in CO_2_ compared to feed 2 and is therefore recycled back to feed 1. The retentates from M3 and M4 are depleted in CO_2_ compared to the respective feeds and are recycled back to M1, as well. The membranes are sized such that the recycling streams have approximately the same composition as the streams to which they are recycled back. The advantage of this design is that no streams of significantly different composition are mixed. If that was the case, energy would be wasted for their prior separation. With the proposed approach, an efficient design is achieved.

| 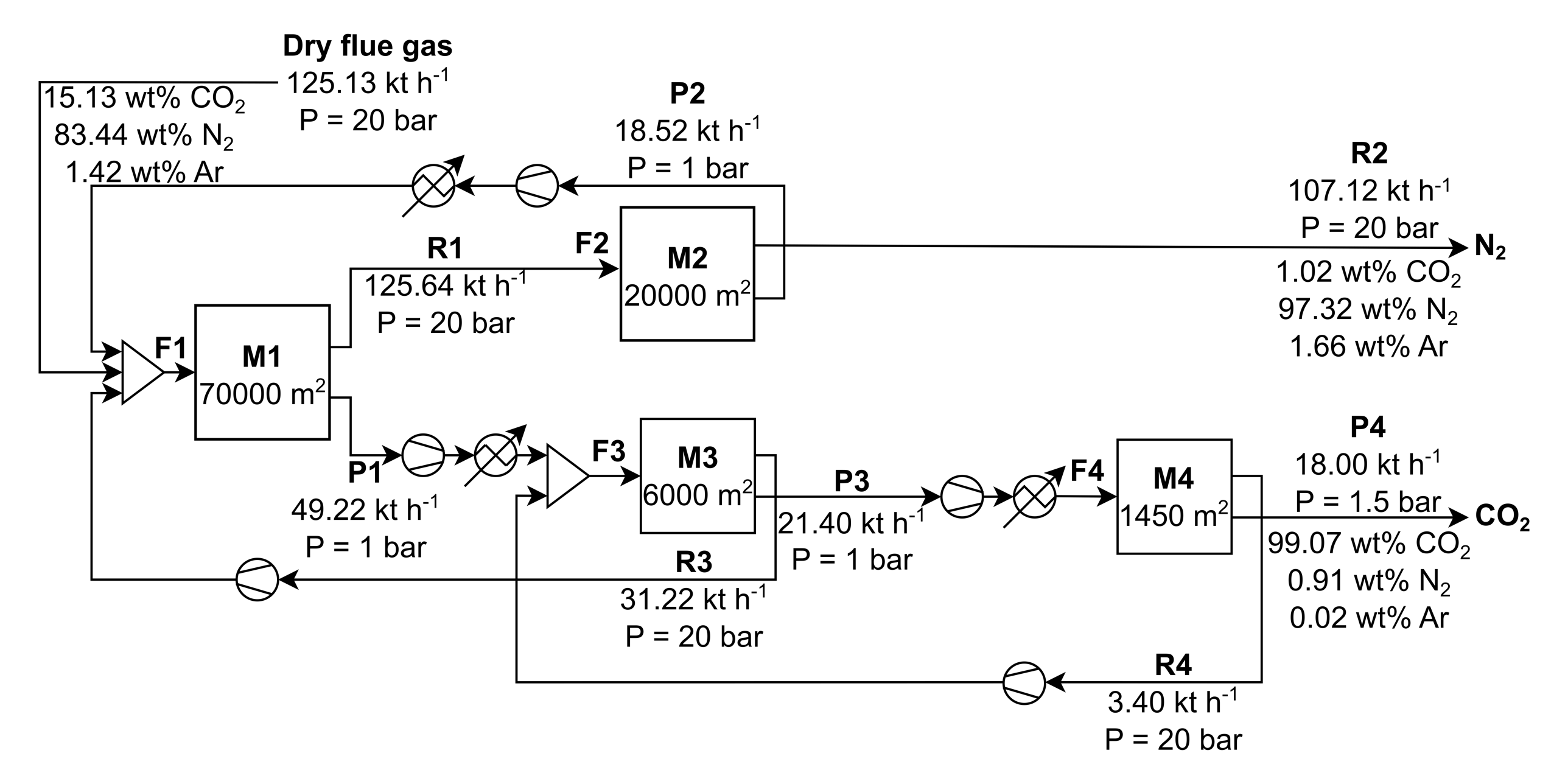 |
| --- |
| **Figure S2.** Flowsheet reporting the membrane module assembly section. The naming convention adopts M for membrane modules, F for their respective feed, R for its retentate, and P for its permeate. |

**Table S4.** Key characteristics of membrane modules used for flue gas separation.

| **Parameter** | **Membrane 1** | **Membrane 2** | **Membrane 3** | **Membrane 4** |
| --- | --- | --- | --- | --- |
| Area (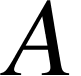) [m^2^] | 70000 | 20000 | 6000 | 1450 |
| Permeate outlet pressure (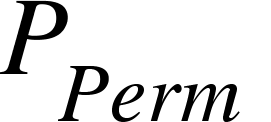) [bar] | 1 | 1 | 1 | 1.5 |
| Feed flow rate (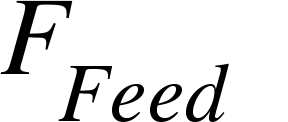) [t h^−1^] | 174.87 | 125.64 | 52.62 | 21.40 |
| Fraction of CO_2_ in feed (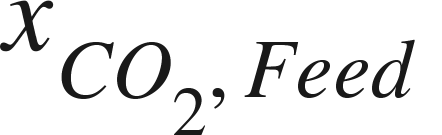) [-] | 0.104 | 0.021 | 0.357 | 0.868 |
| Retentate flow rate (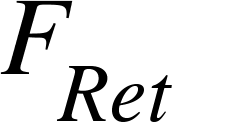) [kt h^−1^] | 125.64 | 107.12 | 31.22 | 3.40 |
| Fraction of CO_2_ in retentate  (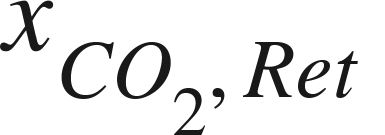) [-] | 0.021 | 0.007 | 0.107 | 0.381 |
| Permeate flow rate (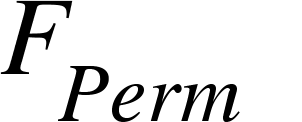) [kt h^−1^] | 49.22 | 18.52 | 21.40 | 18.00 |
| Fraction of CO_2_ in permeate  (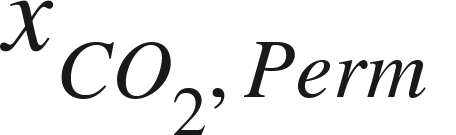) [-] | 0.355 | 0.106 | 0.868 | 0.986 |

- 1. **Water electrolysis**

As Aspen HYSYS^®^ does note provide an electrolyzer model, custom models were developed using ACM. All the electrolysis models follow the same general design (see **Figure S3**). A unit is designed in ACM having one material inlet stream for water or the aqueous mixture and two or three outlet streams in high- and low-temperature electrolysis, respectively. The unreacted water is used to cool the electrolysis stack, avoiding additional cooling. A selection of notable parameters for the three electrolyzer types is provided in **Table S5**.

**Table S5.** Key characteristics of electrolyzers used for H_2_ production.

| **Parameter** | **AEC** | **PEMEC** | **SOEC** |
| --- | --- | --- | --- |
| Reactive area (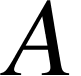) [m^2^] | 300000 | 707500 | 471700 |
| Cell voltage (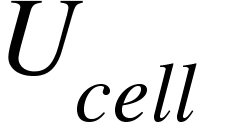) [V] | 1.77 | 1.89 | 1.42 |
| Power (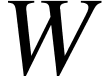) [GW] | 1.37 | 1.34 | 1.00 |
| Feed flow rate (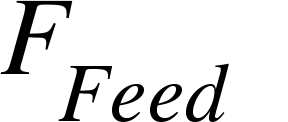) [t h^−1^] | 4504 | 4504 | 270 |
| Temperature (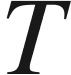) [°C] | 89 | 89 | 1076 |

| 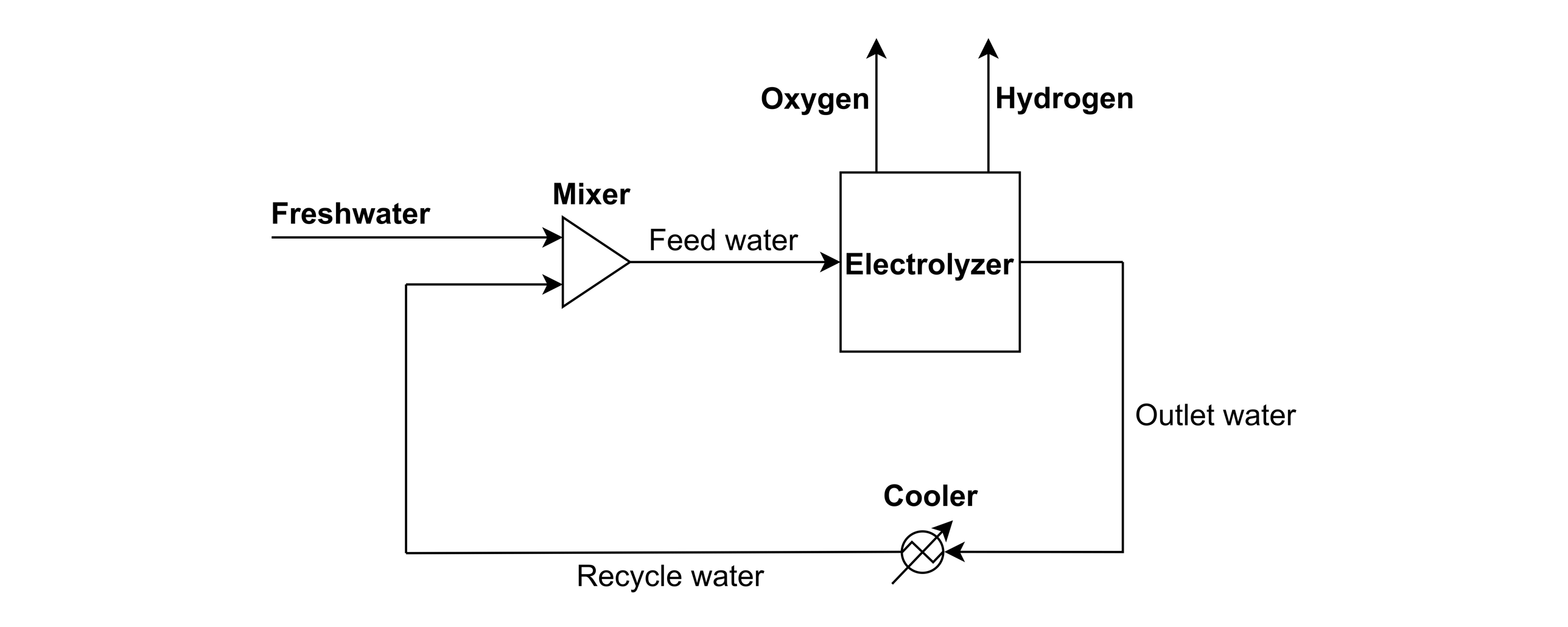 |
| --- |
| **Figure S3.** General structure of the low-temperature water electrolysis section. In the case of SOEC, the recycled water is condensed from the H_2_ outlet stream, there is no cooler after this condensation step, and the feed water is preheated and evaporated. |

The water present in the gaseous outlets of the low-temperature electrolysis is assumed to be at saturation levels. The outlet flow of water is calculated using a mass balance over the entire electrolysis unit. The stoichiometric equation of water electrolysis is given as:

| 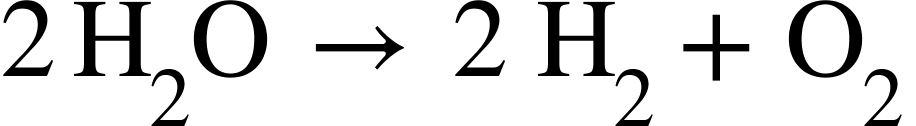 | (Equation S18) |
| --- | --- |

The molar flow rate of the generated product
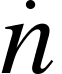
 is connected with the total current
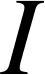
 by Faraday's law of electrolysis:^10–12^

| 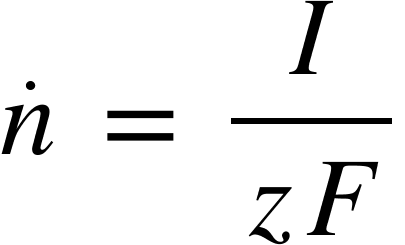 | (Equation S19) |
| --- | --- |

with the Faraday constant
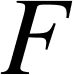
 and the number of electrons per mole of product
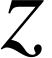
.

The temperature inside the electrolyzer is assumed to be constant throughout the whole unit and equal to the outlet temperature. It is calculated through an energy balance over the entire unit using the enthalpy calculation function provided by ACM and is given by:

| 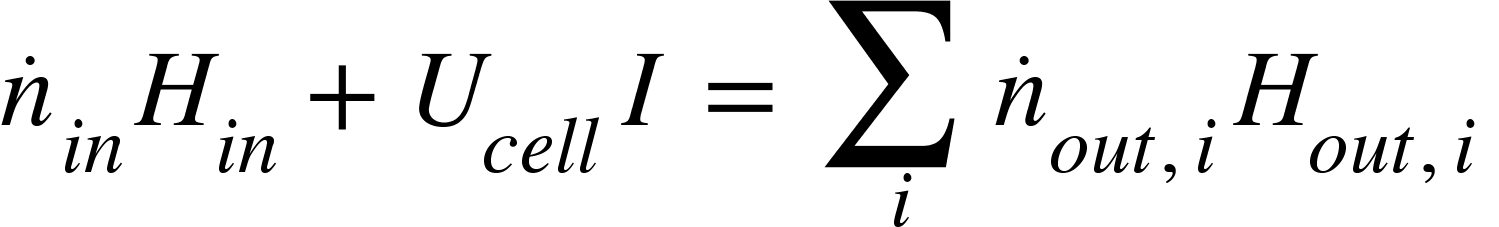 | (Equation S20) |
| --- | --- |


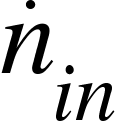
 and
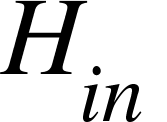
 are the inlet flow rate and molar enthalpy, while
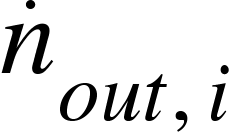
 and
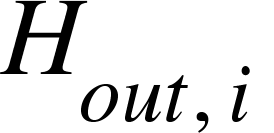
 are the flow rate and molar enthalpy of outlet stream
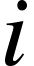
. The cell voltage
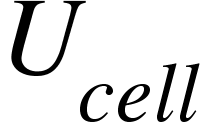
 is calculated as the sum of the equilibrium voltage
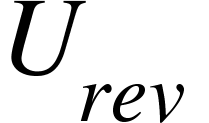
 and the overpotential
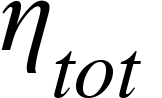
.
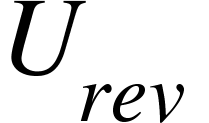
, in volt, is calculated as:

| 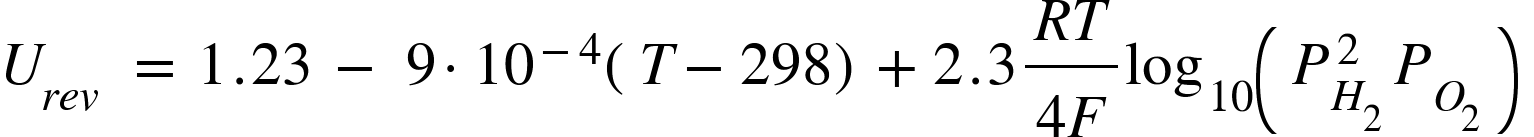 | (Equation S21) |
| --- | --- |

In this equation,
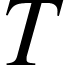
 is given in kelvin and the partial pressures
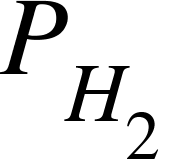
 and
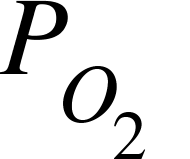
 in bar,
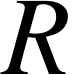
 is the universal gas constant and
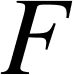
 is the Faraday constant.

The overpotential has three contributions, the ohmic overpotential
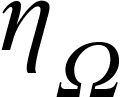
, the activation overpotential
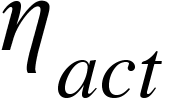
 and the concentration overpotential
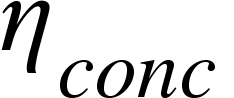
. The latter two each have an anodic and a cathodic contribution. The ohmic overpotential
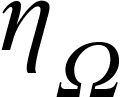
 is caused by the ohmic resistance in the separator between the electrodes and in the electrodes themselves. The activation overpotential
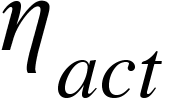
 stems from the kinetic resistance of the electrochemical reactions and the concentration overpotential
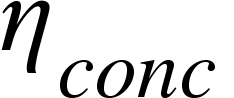
 from local depletion and accumulation of reactants due to mass transfer resistance.

The pressure of PEMEC and SOEC is set to 20 bar, which is in line with the allowed technical specifications for these technologies and reduces the need for downstream pressurization.^13,14^ Alkaline electrolysis is set to work at 5 bar. Higher pressures of 20 bar would technically be possible^13^ but the defined model leads to poor performance at such high pressures.

For alkaline electrolysis, the model uses a closed, semi-empirical equation to calculate the total overpotential , in volt, following the approach presented in Sánchez *et al.*.^12^ In line with the original reference, while the H_2_ diffusion to the O_2_ side is taken into account, the O_2_ diffusion in the opposite direction is considered negligible.

For PEMEC, the concentration overpotential, as well as the crossover of H_2_ and O_2_, are considered negligible. For the ohmic overpotential, only the resistance of the membrane is considered. With this regard, the correlations and the parameters detailed in Ni and coworkers^10^ were adopted. In particular, the integral required to calculate the ohmic resistance was solved by applying the trapezoidal rule and discretizing the interval between the two extremes of the membrane thickness into 50 equally sized intervals. Regarding the activation overpotential, the Butler-Volmer equation was adopted, in line with Ni and coworkers, accounting for the dependence of the kinetic resistance of the reaction on the temperature.^10^

In the case of SOEC, given the drastically different operating conditions, involving temperatures between 600 °C and 1000 °C, the calculation of is adapted:

|  | (Equation S22) |
| --- | --- |

where is given in kelvin and , *i.e.*, the partial pressures at the electrode surfaces, are given in bar.^11^ In line with the study by Ni *et al.*,^11^ O_2_ and H_2_ crossover were neglected. The activation overpotential is calculated as for the PEMEC case,^10^ while the concentration overpotential was calculated using closed expressions that included a cathodic and an anodic contribution. To calculate the cathodic contribution, the effective diffusion coefficient of water was estimated using the Bosanquet formula.^11^ In particular, the H_2_-water gas diffusion coefficient, required to estimate the effective diffusion coefficient of water, was estimated using the Chapman-Enskog theory.^15^ Since in this formula the original reference provided tabulated values for the collision integral , a closed formula was regressed from these values by using a nonlinear fit:

|  | (Equation S23) |
| --- | --- |

where is the Boltzmann constant, the temperature is given in kelvin, and is an energy parameter, calculated as:

|  | (Equation S24) |
| --- | --- |

from the individual molecular parameters.

The values for the regressed parameters in Equation S23 are provided in **Table S6**.

**Table S6.** Parameters regressed for the collision integral closed formula. All the parameters are dimensionless.

| **Parameter** | **Values** |
| --- | --- |
|  | 0.779 |
|  | −0.0057 |
|  | −0.227 |
|  | 0.981 |
|  | −0.874 |

Finally, regarding the ohmic overpotential estimation, only the electrical resistance in the electrolyte was considered, as the electrodes have a much higher conductivity. The relation and the parameters described in Ni et al. were adopted.^11^

- 1. **Sabatier process**

An overall schematic summarizing the modeled SNG production is provided in **Figure S4**.

|  |
| --- |
| **Figure S4.** Schematic representing the modeled SNG production. |

At the beginning, the CO_2_-rich stream is compressed to the operating pressure of 20 bar and mixed with H_2_ already at 20 bar. No drying of H_2_ is necessary, as water is a product of the reaction and water impurities in the feed are thus not an issue. After this step, the mixture is preheated and fed to the first isothermal reactor. The Sabatier reaction follows the relation:

| () | (Equation S25) |
| --- | --- |

For the kinetic model, the reaction is split into two reactions occurring in parallel:^16^

| Methanation |  | (Equation S26) |
| --- | --- | --- |
| Reverse water gas shift |  | (Equation S27) |

The rate model used considers forward and backward reaction for both steps. This allows for reasonable values up to high conversions, which are needed to achieve the desired methane (CH_4_) concentration in the product stream without CO_2_ or H_2_ removal. The model is described by the following equations for a 50% nickel catalyst:

|  | (Equation S28) |
| --- | --- |
|  | (Equation S29) |

where is the reaction rate, are the partial pressures, is the temperature, given in kelvin. The first reaction is the reverse methanation, while the second one is the water gas shift. The kinetic constants are given by Arrhenius-type equations:

|  | (Equation S30) |
| --- | --- |

The adsorption constants and equilibrium constants are described as:

|  | (Equation S31) |
| --- | --- |

The subscript in Equation S30 stands for the reaction, while the subscript in Equation S31 stands for either the species, if the expression describes an adsorption constant, or a reaction, if the expression describes an equilibrium constant. describes either the enthalpy of adsorption, if the expression describes an adsorption constant, or the reaction enthalpy, if the expression involves an equilibrium constant. The parameters associated with these equations are provided in **Table S7**.

**Table S7.** Parameters associated with the Sabatier reaction kinetics.^16,17^

| **Parameter** | **Values** |  | **Parameter** | **Values** |
| --- | --- | --- | --- | --- |
| [mol kg_cat_^−1^ s^−1^] | 1.9×10^10^ |  | [bar^−1^] | 6.12×10^−9^ |
| [kJ mol^−1^] | 103 |  | [kJ mol^−1^] | −82.9 |
| [mol kg_cat_^−1^ s^−1^] | 2.18×10^6^ |  | [bar^−1^] | 6.65×10^−4^ |
| [kJ mol^−1^] | 62 |  | [kJ mol^−1^] | −38.28 |
| [bar^−0.5^] | 5.8×10^−4^ |  | [-] | 1.77×10^5^ |
| [kJ mol^−1^] | −42 |  | [kJ mol^−1^] | 88.68 |
| [bar^−0.5^] | 1.6×10^−2^ |  | [-] | 1.72×10^−2^ |
| [kJ mol^−1^] | −16 |  | [kJ mol^−1^] | 36.6 |
| [bar^−1^] | 8.23×10^−5^ |  | [bar^2^] | 1.225×10^13^ |
| [kJ mol^−1^] | −70.65 |  | [kJ mol^−1^] | −223.1 |

The selected correlations were implemented in a rate-based reactor model in Aspen HYSYS^®^. Reactor size and temperature were selected such that conditions close to the equilibrium are achievable. As shown in Equation S25, the overall enthalpy of reaction is negative, and the number of molecules decreases upon reaction. Thus, a higher conversion can be achieved at lower temperatures and higher pressures. Because of the tradeoff between higher conversion at lower temperatures and smaller reactor sizes at higher ones, a design involving two reactor beds with intercooling was selected. Specifically, in between the two reactors, the reaction mixture is cooled to 30 °C to remove most of the water to pull the equilibrium close to the product side in the second reactor bed. A pressure of 20 bar was set for both beds, and temperatures of 390 °C and 400 °C were selected for the first and the second reactor bed, respectively. The reactor specifications are summarized in **Table S8**. A final conversion of 95.5% and 99.0% with respect to the first reactor inlet are achieved at the end of the first and second reactor bed, respectively.

**Table S8.** Parameters associated with the Sabatier reaction kinetics.

| **Specifications** | **Values** |
| --- | --- |
| [bar] | 20 |
| , first reactor [°C] | 390 |
| , second reactor [°C] | 400 |
| [m^3^] | 1.4 |
| Catalyst loading [kg m^−1^] | 600 |
| Catalyst mass [kg] | 840 |

After the second reactor, the concentrations of H_2_, CO, and CO_2_ are considered low enough to meet the natural gas specifications.^18,19^ N_2_ concentration is controlled *via* the gas separation during the CO_2_ capture before the reaction. While the flash after the second reactor removes most of the water, the SNG must be further dried to reach pipeline specifications. The target maximum concentration, according to pipeline specifications, is considered as 50 ppm. For water removal, TEG dehydration is used, the standard method adopted for natural gas drying, as well.^20^ This dehydration step occurs at the same pressure as the reaction, as already explained in **Section 2.1.1**.

- 1. **Haber-Bosch process**

A schematic of the HB process, adapted from the work by Araújo and Skogestad,^21^ is reported in **Figure S5**. Only minor adaptations to purify the feed from the membrane separation section and polish the final product composition were adopted.

The feed purification was not part of the original source but was here added to achieve the desired feed purity. In fact, impurities of CO_2_, CO, water, and O_2_ need to be below 10 ppm combined.^5^ First, N_2_ from the membrane unit is mixed with H_2_ from the electrolysis unit, both being supplied at 20 bar. The mixture is preheated to 400 °C and passes through a Sabatier reactor to react CO_2_ impurities to CH_4_, since this compound does not interfere with the HB process. The reactor follows the same model as in **Section 2.3**. However, this time an isothermal reactor is selected because of the low CO_2_ concentration and resulting low temperature increase. Any possible CO impurity present in the feed would here be converted to methane, as well. O_2_ removal from traces in the H_2_ feed is not considered explicitly but could be converted catalytically to water as well, as described in **Section 2.1**.

|  |
| --- |
| **Figure S5.** Schematic representing the modeled Haber-Bosch (HB) process. The flowsheet does not include the section of preliminary feed purification, *i.e.*, preliminary methanation and TEG dehydration of the stream. |

The mixture is cooled to 30 °C and dehydrated using TEG as described in the same flue gas preliminary purification section. After this step, it is compressed to 196.3 bar using two compressors in series. Finally, after cooling to 304.2 °C, the stream (“HB feed” in **Figure S5**) is mixed into the reaction loop.

The fresh N_2_-H_2_ mixture is added to the outlet stream of the reactor after that the latter was cooled to 25 °C. This mixed stream is then flashed to remove the liquid ammonia (NH_3_). From the flash gaseous outlet stream a fraction equal to 0.1% is purged, while the remaining amount is fed to the reactor. The purge is assumed to be flared, *i.e.*, converting stoichiometrically the NH_3_, H_2_, and CH_4_ contained in this stream into CO_2_, water, and N_2_. Due to the small flow rate associated with the purge, this step was accounted in terms of final emissions, but excluded from the heat integration. The feed for the reactor is mixed with the recycles originating from NH_3_ cooling and depressurization. After mixing, a compressor increases the pressure to 208 bar. The stream is then split into three feed streams for the three beds of the HB reactor. The first fraction, accounting for 23% of the total reactor feed, flows to the first bed (“Bed 1” in **Figure S5**). The pressure of this stream is reduced to 204 bar by a valve before the reactor inlet and the mixture is preheated to 306.2 °C. The beds are modeled as adiabatic equilibrium reactors. In the first bed, a pressure drop of 1 bar is assumed. The outlet of the first bed is combined with the second feed fraction, containing 13.9% of the total feed. In the second bed (“Bed 2”) the pressure drops to 201.9 bar. Again, the outlet of the second bed is mixed with the third feed fraction, containing the remaining 63.1% of the overall feed, before being fed to the third and final bed (“Bed 3”). Inside the bed the pressure drops to 200.8 bar. After the reactor, the product is cooled to 25 °C in an exchanger with a pressure drop of about 4.5 bar, closing the loop.

After leaving the flash in the reaction loop (“Flash 1” in **Figure S5**), liquid NH_3_ flows through a valve to reduce the pressure to 135 bar. A cooler reduces the temperature to −5 °C with the aid of a refrigeration cycle, working with propylene.^22^ A second flash (“Flash 2”) removes the released gases, which are repressurized and fed back into the reaction loop. After the flash, the NH_3_ pressure is reduced further to 5 bar, whereby the temperature decreases slightly. The stream is then heated to −3 °C. The released gases are removed by a final flash (“Flash 3”), repressurized, and fed back to the reaction loop while the NH_3_ leaves as a finished product.

1. **Further details on the environmental assessment**
   1. **Main assumptions and limitations**

The life cycle assessment (LCA) was performed according to the framework described in the ISO 14040 and ISO 14044 standards.^23,24^

The main assumptions and limitations of the analysis are summarized next:

- Only dispatchable electricity sources, *i.e.*, the electricity mix in all the scenarios, can cover the electricity needs of the main HB process since compressors and pumps cannot operate intermittently.
- We assume that water electrolysis can be powered by dispatchable and non-dispatchable electricity sources.
- Energy storage for non-dispatchable sources powering the electrolyzers was excluded from the analysis. Hence, we assume that the H_2_ produced with intermittent energy sources is temporarily stored in salt caverns, with negligible economic and environmental costs.^25^
- As done in previous works,^26,27^ the O_2_ produced as a by-product of the water electrolysis is assumed to have a zero impact, as the current market could not absorb the amount of O_2_ generated. Furthermore, this O_2_ by-product should be further compressed before being sold, thereby increasing the cost and impact of the main process.
- We assume that the electricity consumed in the background system is modeled with the default activities in Ecoinvent v3.5.^1^ Hence, we only change the electricity of the foreground system while keeping the energy generation activities in the background system unaltered.
- An approach at point of substitution was adopted for the LCI selection.
- For plant construction, an average over several chemical factories was taken as reference. The contribution was scaled according to the assumptions for plant capacity and lifetime (see **Section 3.2** for detailed inventories).^28^
- Different weighting systems could be introduced to penalize the levels of transgression (LTs). Here we assumed, without losing generality, equal weights regardless of the level of transgression and the PB (core/non-core).
- To apply the non-egalitarian downscaling principle, we considered the total Gross Domestic Product (GDP) of Germany. Hence, we impose the corresponding bound *i.e.*, downscaled safe operating space (SOS), on the life cycle impact of the flue gas valorization process, instead of on its production-based impact. Because of the high German GDP, compared to the vast majority of the other countries in the world, such an approach enables Germany to get a higher share of SOS with respect to other countries. Downscaling principles are currently the focus of ongoing research,^29^ and how to avoid double-counting when downscaling the full SOS to different sectors/activities remains open. Here, however, we apply the downscaling only to flue gas valorization.
  1. **Data sources**

This section provides the main data used in the LCA calculations. In **Table S9,** the LCI of the flue gas valorization in terms of high-level sections are reported. including all the dependent sub-processes that were adapted from the original references. The LCI was obtained by combining data from the process model with data from the literature and Ecoinvent v3.5.^1^ Notably, some of the inputs to the foreground system were modeled using additional information from the literature combined with data from Ecoinvent, as described in the tables below. The “Electricity” and “H_2_, wet” activities varies across the different scenarios. A detailed overview of the different electricity scenarios is provided in **Table S10** and **Table S11**. The detailed overview of the different H_2_ scenarios is provided in **Table S12** and **Table S13**. Finally, the BAU, formulated through the system expansion approach, has its inventory presented in **Table S14**.

**Table S9.** LCI of the foreground system in the flue gas valorization scenarios. The main values were taken from the Aspen HYSYS^®^ simulation. The LCI entries of some of the inputs were directly taken from ecoinvent. Other inputs were further disaggregated into their corresponding upstream activities, which were in turn modeled using ecoinvent. The latter activities, *i.e.*, those missing in ecoinvent and requiring a tailored intermediate inventory analysis based on the literature, are labeled with a “*” and further described in the table below. The outputs directly correspond to LCI entries for which characterization factors are available.

| **Process** | **Material/Energy flow** | **Amount** |
| --- | --- | --- |
| Flue gas valorization process | Inputs: |  |
|  | NH_3_, from flue gas valorization [kg]* | 9.38×10^−1^ |
|  | SNG, from flue gas valorization [kg]* | 5.00×10^−1^ |
|  | Flue gas separation [kg]* | 1 |
|  | Cooling water [kg]* | 364 |
|  | Outputs, products: |  |
|  | Flue gas valorization process [kg] | 1 |
| Flue gas separation | Inputs: |  |
|  | H_2_, wet [kg]* | 5.00×10^−3^ |
|  | Polymer membrane [kg]*^,^^[[1]](#footnote-1)^ | 1.72×10^−6^ |
| Flue gas separation | Methanation catalyst [kg]* | 1.43×10^−8^ |
|  | Electricity [kWh] | 8.00×10^−2^ |
|  | Outputs, products: |  |
|  | Flue gas separation [kg] | 1 |
|  | Outputs, emissions/wastes: |  |
|  | Spent catalyst (to treatment for inert material landfill)^31^ [kg] | 1.43×10^−8^ |
|  | Wastewater [m^3^] | 8.49×10^−5^ |
| NH_3_, from flue gas valorization | Inputs: |  |
|  | H_2_, wet [kg]* | 1.82×10^−1^ |
|  | Chemical factory, organics^1^ [p] | 6.67×10^−10^ |
|  | NH_3_ synthesis catalyst^31^ [kg]* | 5.51×10^−5^ |
|  | Electricity [kWh] | 5.32×10^−1^ |
|  | Outputs, products: |  |
|  | NH_3_, from flue gas valorization (>99.5 wt% purity) [kg] | 1 |
|  | Outputs, emissions/wastes: |  |
|  | CO_2_, fossil (to air) [kg] | 2.00×10^−2^ |
|  | Water (to air) [kg] | 4.00×10^−4^ |
|  | Argon (to air) [kg] | 1.04×10^−3^ |
|  | Spent catalyst (to treatment for inert material landfill)^31^ [kg] | 5.51×10^−5^ |
| SNG, from flue gas valorization | Inputs: |  |
|  | H_2_, wet [kg]* | 4.91×10^−1^ |
|  | Chemical factory, organics^1^ [p]* | 6.67×10^−10^ |
|  | Methanation catalyst [kg]* | 1.03×10^−6^ |
|  | Electricity [kWh] | 1.92×10^−1^ |
|  | Outputs, products: |  |
|  | SNG, from flue gas valorization (>94 wt% purity) [kg] | 1 |
| SNG, from flue gas valorization | Outputs, emissions/wastes: |  |
|  | Spent catalyst (to treatment for inert material landfill)^31^ [kg] | 1.03×10^−6^ |
|  | Wastewater [m^3^] | 2.13×10^−3^ |
| Cooling water^1,^^[[2]](#footnote-2)^ | Inputs: |  |
|  | Water, decarbonized, at user, water production and supply [kg] | 3.87×10^−1^ |
|  | Electricity [kWh] | 2.78×10^−5^ |
|  | Outputs, products: |  |
|  | Cooling water [kg] | 1 |
|  | Outputs, emissions/wastes: |  |
|  | Water (to air) [kg] | 3.87×10^−1^ |
| Polymer membrane^27,^^[[3]](#footnote-3)^ | Inputs: |  |
|  | Polydimethylsiloxane [kg] | 2.22×10^−1^ |
|  | Acrylonitrile [kg] | 2.22×10^−1^ |
|  | Polyester resin, unsaturated [kg] | 2.22×10^−1^ |
|  | Polyethylene terephthalate, granulate, amorphous [kg] | 2.05×10^−1^ |
|  | Ethylene glycol [kg] | 1.28×10^1^ |
|  | Outputs, products: |  |
|  | Polymer membrane [kg] | 1 |

| **Process** | **Material/Energy flow** | **Amount** |
| --- | --- | --- |
| Methanation catalyst^27^ | Inputs: |  |
|  | Nickel, 99.5% [kg] | 2.50×10^−1^ |
|  | Zeolite, powder [kg] | 7.50×10^−1^ |
|  | Electricity [kWh] | 4.85×10^−1^ |
|  | Outputs, products: |  |
|  | Methanation catalyst [kg] | 1 |
| NH_3_ synthesis catalyst^27^ | Inputs: |  |
|  | Magnetite [kg] | 9.17×10^−1^ |
|  | Lime [kg] | 3.00×10^−2^ |
|  | Zeolite, powder [kg] | 5.25×10^−2^ |
|  | Electricity [kWh] | 1.78 |
|  | Outputs, products: |  |
|  | NH_3_ synthesis catalyst [kg] | 1 |

**Table S10.** Composition of the 2020 German power mix, adapted from official reports.^2,33–35^ The name of the corresponding entries in ecoinvent is reported.

| **Power source** | **ecoinvent v3.5 entry** | **Share [%]** |
| --- | --- | --- |
| Coal | Electricity, high voltage, lignite | 16.95 |
|  | Electricity, high voltage, hard coal | 7.36 |
| Natural gas | Electricity, high voltage, natural gas, conventional power plant | 12.22 |
| Nuclear | Electricity, high voltage, nuclear, pressure water reactor | 10.80 |
|  | Electricity, high voltage, nuclear, boiling water reactor | 1.69 |
| Photovoltaic | Electricity, low voltage, photovoltaic, 570 kWp open ground installation, multi-Si | 10.48 |
| Wind | Electricity, high voltage, wind, 1-3 MW turbine, onshore | 8.86 |
|  | Electricity, high voltage, wind, >3 MW turbine, onshore | 7.09 |
|  | Electricity, high voltage, wind, 1-3 MW turbine, offshore | 5.5 |
|  | Electricity, high voltage, wind, <1 MW turbine, offshore | 5.32 |
| Biogas | Electricity, high voltage, heat and power co-generation, biogas, gas engine | 7.84 |
| Hydropower | Electricity, high voltage, hydro, run-of-river | 3.78 |
| Biomass | Electricity, high voltage, heat and power co-generation, wood chips, 6667 kW, state-of-the-art 2014 | 2.40 |

**Table S11.** Composition of the wind power mix assumed in this study, and corresponding capacity factors. The shares of offshore and onshore are based on the current proportion of wind power in the 2020 German mix.^2^

| **Label** | **ecoinvent v3.5 entry** | **Share [%]** | **Capacity factor [%]** |
| --- | --- | --- | --- |
| Wind offshore | Electricity, high voltage, wind, 1-3 MW turbine, offshore | 20.55 | 43 |
| Wind onshore | Electricity, high voltage, wind, >3 MW turbine, onshore | 79.45 | 34 |

**Table S12.** LCI associated with H_2_ production in the scenarios based on water electrolysis. The LCI entries of some of the inputs were directly taken from ecoinvent. Other inputs were further disaggregated into their corresponding upstream activities, which were in turn modeled using ecoinvent. The latter activities, *i.e.*, those missing in ecoinvent and requiring an intermediate inventory analysis based on the literature, are labeled with a “*” and further described in the table below. The outputs directly correspond to LCI entries for which characterization factors are available. The electrolyzer areas were adjusted to account for the fraction of active area, according to literature (78.4% for AEC, 66.7% for PEMEC and SOEC).^36^ Moreover, the electrolyzer construction was also scaled up according to the intermittency of the power sources, in order to cover the same annual production. A capacity factor of 100% was assumed for grid power, while a weighted average of wind onshore and offshore was considered for wind-powered scenarios, according to the values provided in **Table S10**.

| **Process** | **Material / Energy flow** | **Amount** | | | | | |
| --- | --- | --- | --- | --- | --- | --- | --- |
| H_2_, wet | Inputs: |  | | | | | |
|  |  | AEC | | PEMEC | | SOEC | |
|  |  | Grid | Wind | Grid | Wind | Grid | Wind |
|  | Electrolyzer construction [m^2^]* | 5.45×10^−5^ | 1.54×10^−4^ | 1.62×10^−4^ | 4.56×10^−4^ | 1.08×10^−4^ | 3.03×10^−4^ |
|  | Electricity [kWh] | 47.76 | | 48.96 | | 36.70 | |
|  | Deionized water, from tap water, at user [kg] | 8.89 | | | | | |
|  | Outputs, products: | | | | | | |
|  | H_2_, wet (>97.5 wt% purity) [kg] | 1 | | | | | |

**Table S13.** LCI associated with H_2_ electrolyzer construction in the scenarios based on water electrolysis, adapted from the work of Zhao and co-authors.^36^ The LCI entries of the inputs were directly taken from ecoinvent. The outputs directly correspond to LCI entries for which characterization factors are available.

| **Process** | **Material / Energy flow** | **Amount** |
| --- | --- | --- |
| Electrolyzer construction – AEC^[[4]](#footnote-4)^ | Inputs: |  |
|  | Nickel, 99.5 wt% [kg]^[[5]](#footnote-5)^ | 1.39×10^1^ |
|  | Polyphenylene sulfide [kg] | 1.94 |
|  | Steel, chromium steel 18/8 [kg] | 4.66 |
|  | Electricity [kWh] | 1.38 |
|  | Outputs, products: |  |
|  | Electrolyzer construction – AEC [m^2^] | 1 |
|  | Outputs, emissions/wastes: |  |
|  | Residue from mechanical treatment, industrial device [kg] | 3.16×10^−1^ |
| Electrolyzer construction – PEMEC^[[6]](#footnote-6)^ | Inputs: |  |
|  | Titanium, primary [kg] | 9.69 |
|  | Platinum [kg] | 4.30×10^−2^ |
|  | Nafion^TM^ cast membrane [kg] | 1.67×10^−1^ |
|  | Synthetic rubber [kg] | 2.10×10^−2^ |
|  | Steel, chromium steel 18/8 [kg] | 2.37 |
|  | Electricity [kWh] | 1.95 |
| Electrolyzer construction – PEMEC^f^ | Outputs, products: |  |
|  | Electrolyzer construction – PEMEC [m^2^] | 1 |
|  | Outputs, emissions/wastes: |  |
|  | Residue from mechanical treatment, industrial device [kg] | 4.97×10^−1^ |
| Electrolyzer construction – SOEC^[[7]](#footnote-7)^ | Inputs: |  |
|  | Zirconium oxide [kg]^[[8]](#footnote-8)^ | 2.83×10^−1^ |
|  | Aluminum oxide [kg] | 3.90×10^−2^ |
|  | Cerium concentrate, 60% cerium oxide [kg] | 1.11×10^−1^ |
|  | Manganese [kg] | 1.10×10^−2^ |
|  | Cobalt [kg] | 1.20×10^−2^ |
|  | Steel, chromium steel 18/8 [kg] | 1.96×10^1^ |
|  | Electricity [kWh] | 5.06 |
|  | Outputs, products: |  |
|  | Electrolyzer construction – SOEC [m^2^] | 1 |
|  | Outputs, emissions/wastes: |  |
|  | Residue from mechanical treatment, industrial device [kg] | 6.63×10^−1^ |

**Table S14.** LCI associated with business-as-usual (BAU). The LCI entries of some of the inputs were directly taken from ecoinvent. Other inputs were further disaggregated into their corresponding upstream activities, which were in turn modeled using ecoinvent. The latter activities, *i.e.*, those missing in ecoinvent and requiring an intermediate inventory analysis based on the literature, are labeled with a “*” and further described in the table below. The outputs directly correspond to LCI entries for which characterization factors are available.

| **Process** | **Material / Energy flow** | **Amount** |
| --- | --- | --- |
| Business-as-usual (BAU) | Inputs: |  |
|  | NH_3_, liquid, production, from steam reforming [kg] | 9.38×10^−1^ |
|  | Natural gas [kg]* | 5.00×10^−2^ |
|  | Outputs, products: |  |
|  | Business-as-usual (BAU) [kg flue gas valorized] | 1 |
|  | Outputs, emissions/wastes: |  |
|  | CO_2_, to air [kg] | 1.39×10^−1^ |
|  | Water, to air [kg] | 4.15×10^−2^ |
| Natural gas^37^ | Inputs: |  |
|  | Natural gas, high pressure (DE), import from Russia [kg] | 4.00×10^−1^ |
|  | Natural gas, high pressure (DE), import from the Netherlands [kg] | 2.90×10^−1^ |
|  | Natural gas, high pressure (DE), import from Norway [kg] | 2.10×10^−1^ |
|  | Natural gas, high pressure (DE), natural gas production [kg] | 7.00×10^−2^ |
|  | Natural gas, high pressure (DE), market for [kg] | 3.00×10^−2^ |
|  | Outputs, products: |  |
|  | Natural gas [kg] | 1 |

1. **Further details on the economic assessment**
   1. **Main assumptions and limitations**

The process model provides the sizes of the equipment units, and the material and energy flows needed in the economic and environmental assessments. The operating expenditures (OPEX) of the flowsheets were estimated using the cost parameters listed in **Tables S15**-**S16**. The parameters associated to the economic assessment of the H_2_ production section are listed in **Table S17**. Additionally, the capital expenditures (CAPEX) were calculated using the correlations and standard economic parameters available in Towler and Sinnott.^38^ All the cost values are expressed in USD_2019_. It was assumed that the total investment occurs in year zero, and the plant operations start in year one.

**Table S15.** Cost parameters used in the OPEX calculations. All costs have been converted to USD_2019_ using the CEPCI.

| **Flow** | **Value** | **Source** |
| --- | --- | --- |
| Natural gas [USD GJ^−1^] | 6 | ^39^ |
| NH_3_ [USD t^−1^] | 350 | ^40–42^ |
| H_2_ [USD t^−1^] | See Table S17 |  |
| Cooling water [USD t^−1^] | 0.036 | ^26^ |
| Catalyst^[[9]](#footnote-9)^ |  |  |
| Methanation [USD kg^−1^] | 50 | ^43^ |
| NH_3_ synthesis [USD kg^−1^] | 2.92 | ^44^ |
| Electricity |  |  |
| Wind [USD MWh^−1^] | 0.0744 | ^45^ |
| 2020 German grid mix [USD MWh^−1^] | 0.0450 | ^46^ |

**Table S16.** Additional economic parameters used in the calculations.

| **Parameter** | **Value** |
| --- | --- |
| Base year | 2019 |
| Project lifetime [y] | 30 |
| Nominal plant availability [h y^−1^] | 8000 |
| Interest rate [%] | 12.0 |
| Maintenance costs (except for electrolyzer sections, fraction of inside battery limit costs of CAPEX)^38^ [%] | 3 |
| Land and insurance costs (fraction of initial CAPEX)^47^ [%] | 2 |
| Labor cost^48^ [USD h^−1^] | 44 |
| Overall number of shift positions assumed for the system [-] | 11 |
| Managements and supervision costs (fraction of the operating labor cost)^38^ [%] | 25 |

**Table S17.** Parameters for the calculation of the H_2_ cost for the scenarios based on water electrolysis.

| **Component** | **AEC** | | **PEMEC** | **SOEC** | |
| --- | --- | --- | --- | --- | --- |
| Nominal input power^49^ [MW] | 100 | | | | |
| Stack lifetime^50^ [h] | 60000 | | 45000 | 25000 | |
| Capital cost^49^ [USD kW^−1^] | 920 | | 1080 | 1670 | |
| Economic parameter | Value | | | | |
| Equipment replacement cost (fraction of initial CAPEX, occurring every time the stack lifetime is reached)^47^^[[10]](#footnote-10)^ [%] | 37% | 39% | | | 38% |
| Other materials costs other than electricity (fraction of initial CAPEX)^13^ [%] | 1.5 | | | | |
| Annual maintenance cost (fraction of initial CAPEX)^13^ [%] | 2 | | | | |

- 1. **Details on Capital Expenditure (CAPEX) calculations**

All the cost values are expressed in USD_2019_. Except for the membrane module, all the other equipment units were costed according to Sinnott and Towler.^38^ All the units were costed according to the general formula:

|  | (Equation S32) |
| --- | --- |

where is the purchased cost, is the sizing factor and and are sizing parameters standardized for each equipment unit. This value is then multiplied by an installation factor to obtain the total investment cost of the equipment unit. The installation factor includes also a location factor, that converts the results based, in terms of location, on the U.S. Gulf Coast, to Germany.

Regarding the membrane modules, the purchased equipment cost of each module is calculated according to the following equation:^51^

|  | (Equation S33) |
| --- | --- |

where is the membrane area, is the feed pressure, and and are cost factors, defined according to the original source. For consistency, the factors proposed in the same source are used to calculate the total capital requirement of the capture system, which includes both direct and indirect costs associated with the installation of the whole capture unit. The so calculated total capital requirement was then updated from the original reference year of the original source to 2019. Given the claimed membrane lifetime higher than 10 years for the Evonik Sepuran^®^ Green technology, no replacement of the component was optimistically assumed.^9^

Regarding the HB reactor, its sizing is based on Araujo and Skogestad.^21^ The reactor cost was approximated as the cost of the pressure vessel and the cost of the catalyst required. About the Sabatier reactor, the vessel itself is not sized, under the assumption that most of its capital cost stems from the tubes required to facilitate heat transfer. Consequently, its cost is indirectly calculated by the cost of the heat exchangers resulting from heat integration of the reactor with other streams. In addition to this indirect costing, the cost of the catalyst was explicitly considered.

1. **Further details on the sensitivity analysis**

Given the strong contribution of H_2_ production on both the environmental and economic performance of the valorization scenarios and the potential variability of technical performance associated with this plant section, a selection of parameters were varied within reasonable boundaries. Furthermore, such analysis can assess the robustness of the results associated with the proposed valorization scenarios. The intervals of variations assumed for each parameter are reported in **Table S18**. The lower and upper bounds for the variations from the base case were estimated based on ranges provided in a selection of references.

For the environmental impacts, the electrolyzer energy consumption was varied under the assumption of it linearly affecting the electricity-related contribution to the total impact. Similarly, the electrolyzer size was varied according to improvements in the current density of the system, as found in the literature,^52^ this having a linear effect on the stack construction environmental contribution.

In terms of economic scope, along with electrolyzer energy consumption and electrolyzer size, the electrolyzer cost was also varied, with a linear effect on the H_2_ CAPEX contribution.^52^ Moreover, for the cases using wind energy, the associated levelized cost of electricity was also varied within realistic limits.^34^

Each parameter was here considered independent from the other ones and linearly affecting the associated impact contributions. Such assumption, while quite realistic for the case of energy consumption and levelized cost of electricity, might be overly optimistic for the case of electrolyzer size and cost, since beyond a certain threshold a difference in current density could indirectly affect the cost per kW of each stack. Nevertheless, the assumption of independence of the different parameters was here considered as a reasonable approximation for the scope of the study.

**Table S18.** Parameters used in the sensitivity and relative variation with respect to the base case for the scenarios based on water electrolysis.

| **Parameter varied** | **Lower bound** | **Upper bound** |
| --- | --- | --- |
| Electrolyzer energy consumption^52^ | −15% | +10% |
| Electrolyzer size^52^ | −50% | +50% |
| Electrolyzer cost^52^ | −50% | +100% |
| Levelized cost of electricity from wind^45^ | −40% | +120% |

1. **Additional results**

We provide next some additional results omitted in the main body of the manuscript due to space limitations.

- 1. **Additional results: environmental analysis, non-egalitarian downscaling approach, and ReCiPe 2016 full endpoint impacts**

**Figure S6** shows the full detailed breakdown of all the impacts associated with Figure 3 in the main manuscript. **Figure S7** provides the full results (for all the scenarios) in terms of the level of transgression related to the PBs “biogeochemical flows – nitrogen” and “freshwater use”. It should be highlighted that, for the latter, most of the impact in the alternative scenarios stem from the assumption of high evaporation rate of the cooling water, which embeds most of the impact. Furthermore, **Figure S8** provides the level of transgression related to the PBs “stratospheric ozone depletion”, “biogeochemical flows – phosphorus”, and “land system change”, while **Figure S9** provides the results for the endpoints of the ReCiPe 2016 life cycle impact methodology. Furthermore, **Figure S10** shows the impact breakdown for H_2_ production related to two major metrics used in this study, namely, climate change – CO_2_ concentration and human health impacts.

|  |
| --- |
| **Figure S6.** Full breakdown of the impacts related to the PBs “climate change – CO_2_ concentration”, “climate change – energy imbalance”, “ocean acidification”, and “terrestrial biosphere integrity” and the ReCiPe 2016 endpoint “human health”, for all the assessed routes. |

|  |
| --- |
| **Figure S7.** Level of transgression and main breakdown (A) and further breakdown (B) of the impacts related to the PBs “biogeochemical flows – nitrogen” and “freshwater use”. |

|  |
| --- |
| **Figure S8.** Level of transgression and main breakdown (A-B) and further breakdown (C-D) of the impacts related to the PBs “stratospheric ozone depletion” and “biogeochemical flows – phosphorus” (A and C), as well as “land system change” (B and D). |

|  |
| --- |
| **Figure S9.** Total impact and main breakdown (A-C) and further breakdown (D-F) for the three endpoint-level categories associated with the ReCiPe 2016 methodology, i.e., human health impacts (A and D), impacts on ecosystems (B and E), and resource scarcity (C and F). |
|  |
| **Figure S10.** Impact breakdown for electrolytic H_2_ production for the major PB indicator in terms of level of transgression, climate change – CO_2_ concentration (A), and for one of the ReCiPe 2016 endpoints, human health impacts (B). The impact breakdown for the other major PB indicators reported in Figure 2 in the main manuscript are here omitted, since they have a similar distribution of the contributions to climate change – CO_2_ concentration. |

- 1. **Additional results: environmental analysis, egalitarian downscaling approach**

This section presents the results obtained applying an alternative egalitarian population-based downscaling approach to the assessed control variables. **Figure S11** provides the full results (for all the scenarios) in terms of the level of transgression related to the PBs indicators “climate change – CO_2_ concentration”, “climate change – energy imbalance”, “ocean acidification”, and “terrestrial biosphere integrity”. Furthermore, **Figure S12** shows the level of transgression associated with the control variables “biogeochemical flows – nitrogen” and “freshwater use”. Finally, **Figure S13** provides the level of transgression related to the PBs “stratospheric ozone depletion”, “biogeochemical flows – phosphorus”, and “land system change”.

|  |
| --- |
| **Figure S11.** Level of transgression and main breakdown of the impacts related to the PBs “climate change – CO_2_ concentration”, “climate change – energy imbalance”, “ocean acidification”, and “terrestrial biosphere integrity” using the egalitarian population-based downscaling approach. |

|  |
| --- |
| **Figure S12.** Level of transgression and main breakdown of the impacts related to the PBs “biogeochemical flows – nitrogen” and “freshwater use” using the egalitarian population-based downscaling approach. |

|  |
| --- |
| **Figure S13.** Level of transgression and breakdown of the impacts related to the PBs “stratospheric ozone depletion” and “biogeochemical flows – phosphorus” (A), as well as “land-system change” (B) using the egalitarian population-based downscaling approach. |

- 1. **Additional results: economic analysis**

The full economic results for all the investigated alternative scenarios are presented in **Figure S14**, while **Figure S15** provides a cost breakdown of the H_2_ cost for the presented alternative scenarios.

|  |
| --- |
| **Figure S14.** (A-B) Total production cost breakdown for the selected alternative scenarios. The main breakdown is provided in the bar plot (A), while a further breakdown is reported in the pie charts (B). The colors show the distribution of economic impacts with respect to the different sections of the plant, while the patterns show the distribution with respect to CAPEX share and several contributors to the OPEX, including H_2_, fixed costs, electricity, and cooling water. |

|  |
| --- |
| **Figure S15.** Total production cost breakdown for the selected scenarios of H_2_ production. |

- 1. **Additional results: sensitivity analysis**

The results associated with the sensitivity analysis for the environmental impacts is provided in **Figure S16**, while **Figure S17** provides the results of the sensitivity analysis associated with the economic impacts.

The ratios in the heatmaps are calculated according to the following equation:

|  | (Equation S34) |
| --- | --- |

where is the input parameter for the base case, is the input parameter after variation, is the output for the base case, and is the output after variation of the considered input parameter.

The values reported in the right-hand side of the figures correspond to the minimum and maximum value of the denominator output ratios in Equation S34 across all the scenarios. One can thus calculate the output absolute values after input variation from the output values for the base case presented in **Figures 2**, **S7**, **S8**, and **S9**.

|  |
| --- |
| **Figure S16.** Results of the sensitivity analysis associated with the environmental analysis.  (A) Ratios of relative change of each output indicator versus relative change of each varied input parameter for each assessed valorization scenario. (B) Relative maximum compounded output variation with respect to the base case across all the assessed scenarios. |

|  |
| --- |
| **Figure S17.** Results of the sensitivity analysis associated with the economic analysis. (A) Ratios of relative change of each output indicator versus relative change of each varied input parameter for each assessed valorization scenario. (B) Relative maximum compounded output variation with respect to the base case across all the assessed scenarios. |

**References**

(1) Wernet, G.; Bauer, C.; Steubing, B.; Reinhard, J.; Moreno-Ruiz, E.; Weidema, B. The Ecoinvent Database Version 3 (Part I): Overview and Methodology. *Int. J. Life Cycle Assess.* **2016**, *21* (9), 1218–1230, DOI 10.1007/s11367-016-1087-8.

(2) Burger, B. *Net Public Electricity Generation in Germany in 2020*; Fraunhofer-Institut für Solare Energiesysteme (ISE), 2021.

https://www.ise.fraunhofer.de/content/dam/ise/en/documents/News/electricity_production_germany_2020.pdf (accessed 2021-11-19).

(3) Al Hashmi, A. B.; Mohamed, A. A. A.; Dadach, Z. E. Process Simulation of a 620 MW-Natural Gas Combined Cycle Power Plant with Optimum Flue Gas Recirculation. *Open J. Energy Effic.* **2018**, *7* (2), 33–52, DOI 10.4236/OJEE.2018.72003.

(4) *Energiemarkt Deutschland 2020*; Bundesverband der Energie- und Wasserwirtschaft, 2020. https://www.bdew.de/service/publikationen/bdew-energiemarkt-deutschland-2020 (accessed 2021-11-19).

(5) Appl, M. Ammonia, 2. Production Processes. In *Ullmann’s Encyclopedia of Industrial Chemistry*; Wiley-VCH Verlag GmbH & Co. KGaA: Weinheim, Germany, 2011, DOI 10.1002/14356007.o02_o11.

(6) Wiesmann, T.; Youn Suh, S.; Kaluza, S.; Lohmann, H.; Zeidler-Fandrich, B. Catalytic Oxygen Removal from Synthetic Coke Oven Gas: A Comparison of Sulfided CoMo/γ‑Al_2_O_3_ and NiMo/γ‑Al_2_O_3_ Catalysts with Pt/γ‑Al_2_O_3_ as Benchmark Catalyst. *Chem. Ing. Tech.* **2020**, *92* (10), 1533–1541, DOI 10.1002/cite.202000067.

(7) Kuhn, A. N.; Chen, Z.; Lu, Y.; Yang, H. Sequential Oxygen Reduction and Adsorption for Carbon Dioxide Purification for Flue Gas Applications. *Energy Technol.* **2019**, *7* (4), 1800917, DOI 10.1002/ente.201800917.

(8) Zanco, S. E.; Pérez-Calvo, J.-F.; Gasós, A.; Cordiano, B.; Becattini, V.; Mazzotti, M. Postcombustion CO_2_ Capture: A Comparative Techno-Economic Assessment of Three Technologies Using a Solvent, an Adsorbent, and a Membrane. *ACS Eng. Au* **2021**, *1* (1), 50–72, DOI 10.1021/acsengineeringau.1c00002.

(9) Evonik. SEPURAN^®^ Green - Membrane technology for efficient biogas upgrading. https://products.evonik.com/assets/44/34/Asset_684434.pdf (accessed 2023-06-02).

(10) Ni, M.; Leung, M. K. H.; Leung, D. Y. C. Electrochemistry Modeling of Proton Exchange Membrane (PEM) Water Electrolysis for Hydrogen Production. In *World Hydrogen Energy Conference 16*; Lyon, June 13-16, 2006.

(11) Ni, M.; Leung, M. K. H.; Leung, D. Y. C. Parametric Study of Solid Oxide Steam Electrolyzer for Hydrogen Production. *Int. J. Hydrog. Energy* **2007**, *32* (13), 2305–2313, DOI 10.1016/j.ijhydene.2007.03.001.

(12) Sánchez, M.; Amores, E.; Abad, D.; Rodríguez, L.; Clemente-Jul, C. Aspen Plus Model of an Alkaline Electrolysis System for Hydrogen Production. *Int. J. Hydrog. Energy* **2020**, *45* (7), 3916–3929, DOI 10.1016/j.ijhydene.2019.12.027.

(13) Bertuccioli, L.; Chan, A.; Hart, D.; Lehner, F.; Madden, B.; Standen, E. *Study on Development of Water Electrolysis in the EU*; Fuel Cells and Hydrogen Joint Undertaking: Lausanne, 2014; pp 1–160.

https://www.fch.europa.eu/sites/default/files/study%20electrolyser_0.pdf (accessed 2021‑11‑19).

(14) Hauch, A.; Küngas, R.; Blennow, P.; Hansen, A. B.; Hansen, J. B.; Mathiesen, B. V.; Mogensen, M. B. Recent Advances in Solid Oxide Cell Technology for Electrolysis. *Science* **2020**, *370* (6513), eaba6118, DOI 10.1126/science.aba6118.

(15) Cussler, E. L. Values of Diffusion Coefficients. In *Diffusion: Mass Transfer in Fluid Systems*; Cambridge Series in Chemical Engineering; Cambridge University Press: Cambridge, 2009; pp 119–122.

(16) Rönsch, S.; Köchermann, J.; Schneider, J.; Matthischke, S. Global Reaction Kinetics of CO and CO_2_ Methanation for Dynamic Process Modeling. *Chem. Eng. Technol.* **2016**, *39* (2), 208‑218, DOI 10.1002/ceat.201500327.

(17) Curto, D.; Martín, M. Renewable Based Biogas Upgrading. *J. Clean. Prod.* **2019**, *224*, 50‑59, DOI 10.1016/j.jclepro.2019.03.176.

(18) TC Energy. Gas Quality Specifications - TC Energy and other pipelines. http://www.tccustomerexpress.com/docs/Gas_Quality_Specifications_Fact_Sheet.pdf (accessed 2023-06-01).

(19) del Álamo, J. NG/Biomethane Fuel Specification in Europe, 2013. https://wiki.unece.org/download/attachments/5802786/GFV+26-05e+rev+1.pdf (accessed 2023‑06‑01).

(20) Anyadiegwu, C. I. C.; Kerunwa, A.; Oviawele, P. Natural Gas Dehydration Using Triethylene Glycol (TEG). *Pet. Coal* **2014**, *56* (4), 407–417.

(21) Araújo, A.; Skogestad, S. Control Structure Design for the Ammonia Synthesis Process. *Comput. Chem. Eng.* **2008**, *32* (12), 2920–2932, DOI 10.1016/j.compchemeng.2008.03.001.

(22) Luyben, W. L. Refrigerant Selection for Different Cryogenic Temperatures. *Comput. Chem. Eng.* **2019**, *126*, 241–248, DOI 10.1016/j.compchemeng.2019.03.030.

(23) International Organization for Standardization. *ISO 14040:2006 - Environmental Management - Life Cycle Assessment: Principles and Framework*; ISO, 2006.

(24) International Organization for Standardization. *ISO 14044:2006 - Environmental Management - Life Cycle Assessment: Requirements and Guidelines*; ISO, 2006.

(25) Le Duigou, A.; Bader, A. G.; Lanoix, J. C.; Nadau, L. Relevance and Costs of Large Scale Underground Hydrogen Storage in France. *Int. J. Hydrog. Energy* **2017**, *42* (36), 22987‑23003, DOI 10.1016/j.ijhydene.2017.06.239.

(26) Ioannou, I.; D’Angelo, S. C.; Martín, A. J.; Pérez-Ramírez, J.; Guillén-Gosálbez, G. Hybridization of Fossil- and CO_2_-Based Routes for Ethylene Production Using Renewable Energy. *ChemSusChem* **2020**, *13* (23), 6370–6380, DOI 10.1002/cssc.202001312.

(27) D’Angelo, S. C.; Cobo, S.; Tulus, V.; Nabera, A.; Martín, A. J.; Pérez-Ramírez, J.; Guillén‑Gosálbez, G. Planetary Boundaries Analysis of Low-Carbon Ammonia Production Routes. *ACS Sustain. Chem. Eng.* **2021**, *9* (29), 9740–9749, DOI 10.1021/acssuschemeng.1c01915

(28) Althaus, H.; Chudacoff, M.; Hischier, R.; Jungbluth, N.; Osses, M.; Primas, A.; Hellweg, S. *Life Cycle Inventories of Chemicals. Ecoinvent Report No.8, v2.0.*; Swiss Centre for Life Cycle Inventories, Swiss Federal Laboratories for Materials Testing and Research (EMPA): Dübendorf, 2007; pp 1–957.

(29) Ryberg, M. W.; Andersen, M. M.; Owsianiak, M.; Hauschild, M. Z. Downscaling the Planetary Boundaries in Absolute Environmental Sustainability Assessments – A Review. *J. Clean. Prod.* **2020**, *276*, 123287, DOI 10.1016/j.jclepro.2020.123287.

(30) Shishatskii, A. M.; Yampol’skii, Yu. P.; Peinemann, K.-V. Effects of Film Thickness on Density and Gas Permeation Parameters of Glassy Polymers. *J. Membr. Sci.* **1996**, *112* (2), 275‑285, DOI 10.1016/0376-7388(95)00301-0.

(31) EIPPCB. *Reference Document on Best Available Techniques for the Manufacture of Large Volume Inorganic Chemicals - Ammonia, Acids and Fertilisers*; Institute for Prospective Technological Studies: Sevilla, 2007.

http://eippcb.jrc.ec.europa.eu/reference/lvic-aaf.html.

(32) Castellani, B.; Rinaldi, S.; Morini, E.; Nastasi, B.; Rossi, F. Flue Gas Treatment by Power‑to‑Gas Integration for Methane and Ammonia Synthesis – Energy and Environmental Analysis. *Energy Convers. Manag.* **2018**, *171*, 626–634, DOI 10.1016/j.enconman.2018.06.025.

(33) Geschäftsstelle der Arbeitsgruppe Erneuerbare Energien-Statistik (AGEE-Stat) am Umweltbundesamt. *Renewable energies in Germany - Data on the development in 2020*; INIS‑DE‑3160; Umweltbundesamt: Dessau-Roßlau, 2021; p 28.

https://www.umweltbundesamt.de/publikationen/erneuerbare-energien-in-deutschland-2020.

(34) Durstewitz, M.; Berkhout, V.; Faulstich, S.; Görg, P.; Große, L.; Hahn, B.; Lutz, M.-A.; Mayer, J.; Pfaffel, S.; Rehwald, F.; Spriestersbach, S. *Wind Energy Report Germany 2018*; Mackensen, R., Series Ed.; Fraunhofer-Institut Für Energiewirtschaft und Energiesystemtechnik: Kassel, 2019.

https://windmonitor.iee.fraunhofer.de/opencms/export/sites/windmonitor/img/Windmonitor-2018/WERD_2018.pdf.

(35) International Atomic Energy Agency. Country Nuclear Power Profiles - Germany 2022. https://cnpp.iaea.org/countryprofiles/Germany/Germany.htm (accessed 2023-06-02).

(36) Zhao, G.; Kraglund, M. R.; Frandsen, H. L.; Wulff, A. C.; Jensen, S. H.; Chen, M.; Graves, C. R. Life Cycle Assessment of H_2_O Electrolysis Technologies. *Int. J. Hydrog. Energy* **2020**, *45* (43), 23765–23781, DOI 10.1016/j.ijhydene.2020.05.282.

(37) Wingas. Sichere Energie für Europa, Unsere Erdgasversorgung basiert auf mehreren Säulen.

https://www.wingas.com/rohstoff-erdgas/woher-bezieht-europa-erdgas.html (accessed 2021‑07‑24).

(38) Towler, G.; Sinnott, R. *Chemical Engineering Design. Principles, Practice and Economics of Plant and Process Design*, 2^nd^ ed.; Butterworth-Heinemann: Oxford, 2013.

(39) Natural Gas Price Forecast: 2021, 2022 and Long Term to 2050. Knoema. https://knoema.de//ncszerf/natural-gas-price-forecast-2021-2022-and-long-term-to-2050 (accessed 2023-06-02).

(40) *AMIS Market Monitor, Issue n. 45*; 45; Food and Agriculture Organization: Rome, 2017. https://www.amis‑outlook.org/fileadmin/user_upload/amis/docs/Market_monitor/AMIS_Market_Monitor_Issue_45.pdf (accessed 2023-06-02).

(41) *AMIS Market Monitor, Issue n. 67*; 67; Food and Agriculture Organization: Rome, 2019. https://www.amis‑outlook.org/fileadmin/user_upload/amis/docs/Market_monitor/AMIS_Market_Monitor_Issue_67.pdf (accessed 2023-06-02).

(42) *AMIS Market Monitor, Issue n. 89*; 89; Food and Agriculture Organization: Rome, 2021. https://www.amis‑outlook.org/fileadmin/user_upload/amis/docs/Market_monitor/AMIS_Market_Monitor_Issue_89.pdf (accessed 2023-06-02).

(43) Sun, D.; Simakov, D. S. A. Thermal Management of a Sabatier Reactor for CO_2_ Conversion into CH_4_: Simulation-Based Analysis. *J. CO_2_ Util.* **2017**, *21*, 368–382, DOI 10.1016/j.jcou.2017.07.015.

(44) Fernández-Dacosta, C.; Shen, L.; Schakel, W.; Ramirez, A.; Kramer, G. J. Potential and Challenges of Low-Carbon Energy Options: Comparative Assessment of Alternative Fuels for the Transport Sector. *Appl. Energy* **2019**, *236*, 590–606, DOI 10.1016/j.apenergy.2018.11.055.

(45) Kost, C.; Jülch, V.; Nguyen, H.-T.; Shammugam, S.; Schlegl, T. *Stromgestehungskosten erneuerbare Energien*; Fraunhofer-Institut für Solare Energiesysteme (ISE): Freiburg, 2018. https://www.ise.fraunhofer.de/content/dam/ise/de/documents/publications/studies/DE2018_ISE_Studie_Stromgestehungskosten_Erneuerbare_Energien.pdf.

(46) Durchschnittliche Börsenstrompreise | Energy-Charts.

https://energy‑charts.info/charts/price_average/chart.htm?l=de&c=DE&interval=year&year=2018&nominal_real=nominal&chartColumnSorting=default (accessed 2023-06-02).

(47) Nguyen, T.; Abdin, Z.; Holm, T.; Mérida, W. Grid-Connected Hydrogen Production via Large-Scale Water Electrolysis. *Energy Convers. Manag.* **2019**, *200*, 112108, DOI 10.1016/j.enconman.2019.112108.

(48) Labor cost for manufacturing in European countries 2020. Statista. https://www.statista.com/statistics/950053/labor-cost-for-manufacturing-in-european-countries (accessed 2023-06-02).

(49) Böhm, H.; Zauner, A.; Rosenfeld, D. C.; Tichler, R. Projecting Cost Development for Future Large-Scale Power-to-Gas Implementations by Scaling Effects. *Appl. Energy* **2020**, *264*, 114780, DOI 10.1016/j.apenergy.2020.114780.

(50) Graf, F. *Innovative Large-Scale Energy Storage Technologies and Power-to-Gas Concepts after Optimisation - Roadmap for Large-Scale Storage Based PtG Conversion in the EU up to 2050*; DVGW Deutscher Verein des Gas- und Wasserfaches e.V. Technisch-wissenschaftlicher Verein: Bonn, 2020.

https://www.storeandgo.info/fileadmin/dateien/STORE_GO_power_to_gas_roadmap_update.pdf (accessed 2023-06-02).

(51) Giordano, L.; Roizard, D.; Bounaceur, R.; Favre, E. Evaluating the Effects of CO_2_ Capture Benchmarks on Efficiency and Costs of Membrane Systems for Post-Combustion Capture: A Parametric Simulation Study. *Int. J. Greenh. Gas Control* **2017**, *63*, 449–461, DOI 10.1016/j.ijggc.2017.05.002.

(52) Schmidt, O.; Gambhir, A.; Staffell, I.; Hawkes, A.; Nelson, J.; Few, S. Future Cost and Performance of Water Electrolysis: An Expert Elicitation Study. *Int. J. Hydrog. Energy* **2017**, *42* (52), 30470–30492, DOI 10.1016/j.ijhydene.2017.10.045.

1. The mass per area of the membranes was calculated from the fiber dimensions, assuming a mass density of 0.9 g cm^−3^, which is an average for polymer membranes.^30^ [↑](#footnote-ref-1)
2. An overall pressure drop in the cooling water cycle of 1 bar is assumed. 38.73% is assumed to be evaporated in the cooling tower as the water passes through. The cooling water is supposed to be supplied at 10 °C and heated up to 25 °C. [↑](#footnote-ref-2)
3. In absence of a detailed inventory for the commercial membrane considered for the model, a Polyactive^®^ membrane was considered for the life cycle assessment, as an approximation, with no membrane replacement through the plant lifetime. Given the low relative impact of this module on the assessed indicators, in agreement with previous studies,^32^ this assumption was not considered as restrictive. [↑](#footnote-ref-3)
4. Zirfon and nickel sulfide coating were neglected, for absence of corresponding entries in ecoinvent and negligible contribution to the total mass of the electrolytic cell (<1%). [↑](#footnote-ref-4)
5. All nickel products in the original source were considered as “Nickel, 99.5 wt%”. [↑](#footnote-ref-5)
6. The iridium contribution present in the original source is here neglected. Such an assumption is partially due to the small mass contribution of such a material (<1% of the total, lower than one third of the platinum amount), and partially because iridium extraction is part of the platinum extraction in ecoinvent. Ink material and carbon paper were also neglected, given the low mass contribution (<3% of the total) and absence of corresponding ecoinvent entries. [↑](#footnote-ref-6)
7. Lanthanum strontium cobalt ferrite, yttrium oxide, tape casting slurry, and glass-ceramic were neglected, given the absence of corresponding entries in ecoinvent and the low associated relative impact contribution in the original study. CGO10 and Mn_1_∙5CO_1_∙5O_4_ were split into individual elements. [↑](#footnote-ref-7)
8. Yttria stabilized zirconia (YSZ) and YSZ on nickel were approximated with zirconia. [↑](#footnote-ref-8)
9. The catalysts are assumed to be refilled once every 5 years. [↑](#footnote-ref-9)
10. Costs for stack replacement are not adjusted for different capacity factors, as the lower utilization should increase stack lifetime, balancing out the larger number of stacks required. [↑](#footnote-ref-10)
